# Lipidomics of infant mesenchymal stem cells associate with the maternal milieu and child adiposity

**DOI:** 10.1172/jci.insight.180016

**Published:** 2024-09-03

**Authors:** Lauren E. Gyllenhammer, Vincent Zaegel, Allison M. Duensing, Manoel E. Lixandrao, Dana Dabelea, Bryan C. Bergman, Kristen E. Boyle

**Affiliations:** 1 Department of Pediatrics, University of California, Irvine, School of Medicine, Irvine, California, USA.; 2 Department of Pediatrics, Section of Nutrition, University of Colorado Anschutz Medical Campus, Aurora, Colorado, USA.; 3 The Lifecourse Epidemiology of Adiposity and Diabetes (LEAD) Center, Aurora, Colorado, USA.; 4 Department of Epidemiology, Colorado School of Public Health,; 5 Department of Pediatrics, and; 6 Department of Endocrinology, Metabolism, and Diabetes, University of Colorado Anschutz Medical Campus, Aurora, Colorado, USA.

**Keywords:** Development, Metabolism, Fatty acid oxidation, Human stem cells, Obesity

## Abstract

Our objective was to interrogate mesenchymal stem cell (MSC) lipid metabolism and gestational exposures beyond maternal body mass that may contribute to child obesity risk. MSCs were cultured from term infants of mothers with obesity (*n* = 16) or normal weight (*n* = 15). In MSCs undergoing myogenesis in vitro, we used lipidomics to distinguish phenotypes by unbiased cluster analysis and lipid challenge (24-hour excess fatty acid [24hFA]). We measured MSC AMP-activated protein kinase (AMPK) activity and fatty acid oxidation (FAO), and a composite index of maternal glucose, insulin, triglycerides, free fatty acids, TNF-α, and high-density lipoprotein and total cholesterol in fasting blood from mid and late gestation (~17 and ~27 weeks, respectively). We measured child adiposity at birth (*n* = 29), 4–6 months (*n* = 29), and 4–6 years (*n* = 13). Three MSC clusters were distinguished by triacylglycerol (TAG) stores, with greatest TAGs in Cluster 2. All clusters increased acylcarnitines and TAGs with 24hFA, although Cluster 2 was more pronounced and corresponded to AMPK activation and FAO. Maternal metabolic markers predicted MSC clusters and child adiposity at 4–6 years (both highest in Cluster 3). Our data support the notion that MSC phenotypes are predicted by comprehensive maternal metabolic milieu exposures, independent of maternal BMI, and suggest utility as an at-birth predictor for child adiposity, although validation with larger longitudinal samples is warranted.

## Abstract 2

Infant mesenchymal stem cells (MSC) phenotype differs based on glycerolipids. This MSC phenotype is predicated by the maternal metabolic milieu and prospectively associates with child adiposity.

## Graphical abstract

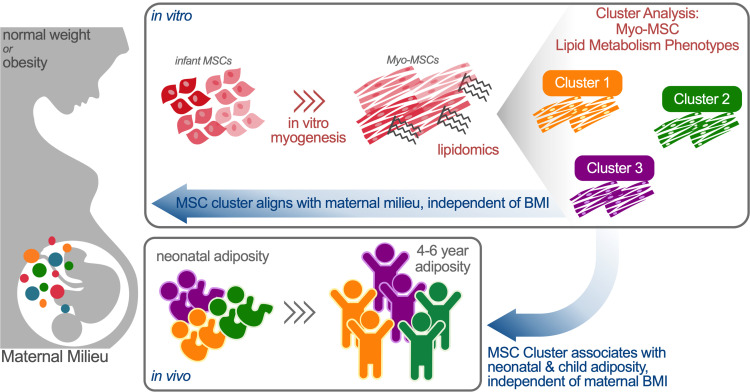

## Introduction

It is evident from the robust body of preclinical animal model and observational human studies that the intrauterine environment is an important contributor to offspring obesity and metabolic disease. Infants exposed to maternal obesity in utero have greater adiposity at birth (1, 2) and childhood (3, 4). However, maternal obesity is an imprecise predictor for offspring adiposity and the specific exposures contributing to future disease risk and the pathways by which this occurs are not well understood in humans.

To address these gaps, we have employed a human infant umbilical cord–derived mesenchymal stem cell (MSC) model to investigate multiple metabolic features of the offspring, including adipogenic propensity, lipid content and metabolism, and metabolic flexibility in association with intrauterine exposures (5, 6). In utero, fetal MSCs are progenitors for mesodermal tissues, including adipose and skeletal muscle, and MSC progenitors are retained in these developed tissues for postnatal growth and repair across the lifespan (e.g., adipose-derived stem cells, satellite cells). MSCs can be readily isolated and cultured from umbilical cord tissue (i.e., Wharton’s jelly), supporting their use as an ethical and feasible tool in studies of human infants. Although our prior data investigating differences between MSCs from infants of mothers with normal weight (NW-MSCs) or obesity (Ob-MSCs) demonstrate remarkable consistency with animal models of maternal obesity (7, 8), we highlight 2 general conclusions from this work. First, MSC metabolic activity does not explicitly correspond to maternal obesity. Rather, maternal metabolic health may be more important in transmitting poor metabolic phenotype to offspring than maternal weight status per se (6, 9, 10). In fact, although Ob-MSCs generally store more lipid than NW-MSCs (6), we have identified divergence among Ob-MSCs for fatty acid oxidation (FAO) (6) and AMP-activated protein kinase (AMPK) activation in response to excess lipid exposure (6, 9), which appears to track with differences in maternal metabolic markers, such as free fatty acids (FFAs). Second, we note that metabolism and storage of lipids are repeatedly identified as important metabolic features intrinsic to the MSCs (5, 6, 9, 10); however, higher lipid storage in MSC-derived myocytes and adipocytes does not necessarily associate with metabolic derangement in these cells. For example, we recently showed that greater lipid stores in MSC-derived myocytes is correlated with greater MSC insulin sensitivity (10). Increasing evidence suggests that the type and pattern of accumulated lipid species may be more consequential to metabolic health than simpler measurements of total lipids (11–14).

The goal of this study was to deeply interrogate MSC lipid metabolism phenotypes, which may help to more precisely define gestational exposures contributing to subsequent child obesity risk, independent of maternal BMI. We harnessed comprehensive MSC lipid phenotyping using quantitative lipidomics, assessed after 21 days of myogenesis, as a cumulative index of the lipid processing and handling across this time period. We used machine learning–based clustering analysis of stored glycerolipids (triacylglycerols [TAGs] and diacylglycerols [DAGs]) to distinguish MSC lipid phenotypes. We hypothesized that these MSC lipid clusters would associate with MSCs’ ability to mobilize lipids and tested this through changes in lipid species, AMPK activation, and FAO measurement in the context of 24-hour lipid challenge studies. We next hypothesized these MSC clusters would be associated with circulating maternal metabolic health measures and longitudinal measures of child adiposity, independent of maternal prepregnancy BMI (ppBMI).

## Results

### K-means clustering of TAG and DAG species reveals distinct MSC phenotypes.

**Figure 1:**
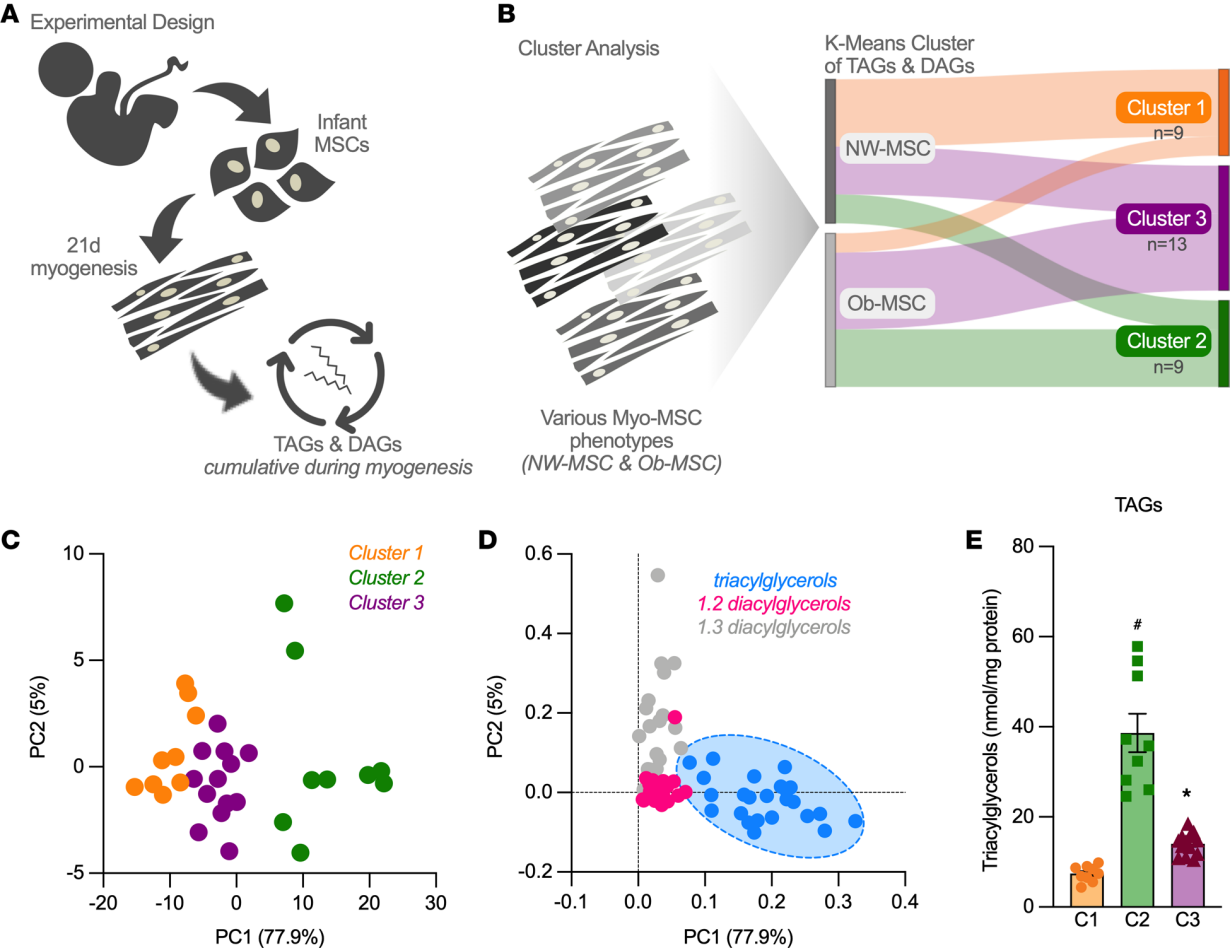
K-means clustering of TAG and DAG species reveals distinct MSC phenotypes. We performed K-means 3-group cluster analysis with all myogenesis-differentiated MSC samples for TAG and DAG species. (**A**) Experimental design is shown. (**B**) Sankey plot shows distribution of NW- and Ob-MSCs to the 3 MSC clusters (Cluster 1, *n* = 9; Cluster 2, *n* = 9; Cluster 3, *n* = 13). (**C**) Principal component analysis shows that component 1 (PC1) accounts for nearly 80% of the variance of the TAG and DAG phenotype and loading plots (**D**) show this is driven by differences in TAG species. (**E**) The sum of all TAG species is shown for the 3 clusters (data are mean ± SEM). ^#^*P* < 0.05, indicates significant difference from Clusters 1 and 3; *P < 0.05, indicates significant difference from Clusters 1 and 2; both by 1-way ANOVA with Kruskal-Wallis post hoc analysis. TAG, triacylglycerols; DAG, diacylglycerols.

Our a priori hypothesis for this study was that MSC lipid metabolism phenotype is defined by the type and pattern of glycerolipids (TAG, DAG) over the course of myogenesis, and by the ability to respond to a lipid challenge. To test the first part of this hypothesis, we first differentiated MSCs into myotubes and used quantitative lipidomics to measure glycerolipid species (Figure 1A). We performed K-means cluster analysis on TAG and DAG species of all MSC samples, regardless of maternal BMI category. Clustering revealed 3 groups (Cluster 1 [*n* = 9], Cluster 2 [*n* = 9], and Cluster 3 [*n* = 13]), each containing a mix of NW- and Ob-MSCs (Figure 1B). Partial least-squares discriminant analysis (PLS-DA) revealed that component 1 explained nearly 80% of the variance of MSC lipid phenotype (Figure 1C) and was largely driven by TAG species (Figure 1D). Component 2 was largely driven by 1,3-DAGs but explained only approximately 5% of the variance (Figure 1D). We confirmed stark differences in the sum of TAG species between the clusters (Figure 1E).

Participant characteristics based on cluster and maternal BMI are shown in Table 1 and Supplemental Table 1, respectively (supplemental material available online with this article; https://doi.org/10.1172/jci.insight.180016DS1). Clusters did not differ based on maternal age, gestational age at delivery, gestational weight gain, or infant sex. ppBMI tended to be lower in Cluster 1 compared with Clusters 2 and 3 (*P* = 0.07 and *P* = 0.13 in Cluster 1 versus Clusters 2 and 3, respectively), and clusters varied by parity, where Clusters 1 and 3 had more primiparous pregnancies than Cluster 2 (*P* = 0.02). Markers of myogenic differentiation and characteristics did not differ by cluster (Supplemental Table 3).

### Maternal metabolic milieu predicts MSC cluster, which in turn, predicts offspring adiposity.

**Figure 2:**
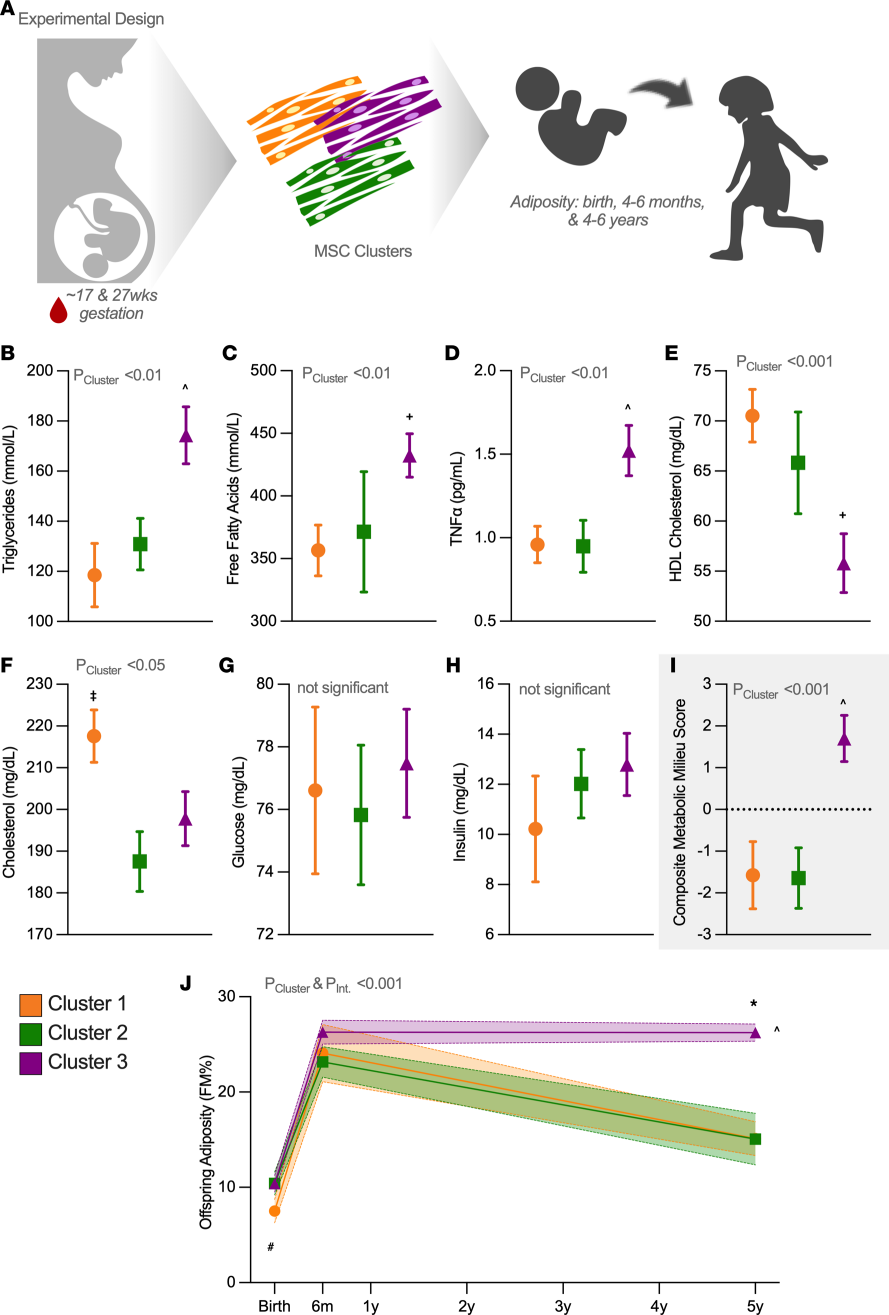
MSC clusters track with maternal and offspring metabolic characteristics. (**A**) We assessed maternal serum at mid and late gestation (~17 and ~27 weeks of gestation) and report the GEE-modeled mean ± SEM across gestation for fasting triglycerides (**B**), free fatty acids (**C**), TNF-α (**D**), HDL-cholesterol (**E**), total cholesterol (**F**), glucose (**G**), and insulin (**H**). Cluster 1, *n* = 9; Cluster 2, *n* = 9; Cluster 3, *n* = 13. (**I**) We then calculated a composite metabolic milieu score (additive *z* score of all individual measures). (**J**) We measured child adiposity at birth (Cluster 1, *n* = 8; Cluster 2, *n* = 8; Cluster 3, *n* = 13), 4–6 months (Cluster 1, *n* = 8; Cluster 2, *n* = 8; Cluster 3, *n* = 13), and 4–6 years (Cluster 1, *n* = 4; Cluster 2, *n* = 4; Cluster 3, *n* = 5) in children from the 3 clusters. We analyzed data using a population-averaged GEE and adjusted all models for maternal age, parity, ppBMI, and infant sex. We additionally adjusted maternal trait models for gestational age at blood draw and child adiposity model for child age at scan. Data are mean ± SEM. ^Cluster 3 different from Clusters 1 and 2, ^+^Cluster 3 different from Cluster 1, ^‡^Cluster 1 different from Clusters 2 and 3, ^#^Cluster 3 tends higher than Cluster 1 at birth (*P* = 0.09), *Cluster 3 higher than Clusters 1 and 2 at 4–6 years. *P* values listed in the figure are for main effects of the analysis (e.g., effect of cluster, interaction), *P* values listed in the legend, indicated by symbols in the panels, refer to the post hoc tests (least-squares means). Data are mean ± SEM.

Using repeated-measure modeling, we next tested whether maternal metabolic characteristics in mid and late gestation predicted MSC cluster (Figure 2A). Given the cluster differences noted in Table 1, we adjusted for ppBMI and parity in addition to our a priori covariates (maternal age, gestational age at blood draw, and infant sex). Maternal triglycerides (throughout the paper we refer to maternal circulating triglycerides as “triglycerides,” while we refer to the MSC triacylglycerols as “TAGs” to avoid confusion with the circulating lipids), high-density lipoprotein (HDL) cholesterol, total cholesterol, FFAs, and tumor necrosis factor α (TNF-α) varied by cluster (*P* < 0.05; Figure 2, B–F), which was consistent across gestation (no interaction with time of gestation). Maternal insulin or glucose did not differ between clusters (Figure 2, G and H). Concordance among these metabolic components underscores the concept that multiple gestational milieu factors may impact offspring outcomes. Therefore, we calculated a composite index of maternal metabolic milieu (additive *z* score of all traits) to broadly assess these exposures. This metabolic milieu score was lower in Clusters 1 and 2 compared with Cluster 3 (*P* < 0.0001; Figure 2I), supporting a combined and additive effect of the maternal milieu on offspring MSC phenotype.

We then tested whether child MSC cluster predicted child adiposity (percentage fat mass [%FM]) from birth to 4–6 years of age. Adiposity trended higher in Cluster 3 compared with Cluster 1 at birth (*P* = 0.09), but by 4–6 years, children from Cluster 3 exhibited higher %FM relative to children from Clusters 1 and 2 (*P* < 0.001, *P*_interaction_ < 0.001, *n* = 13; Figure 2J).

### Acylcarnitines, TAGs, and 1,2-DAGs robustly change in response to lipid challenge in all MSC clusters.

**Figure 3:**
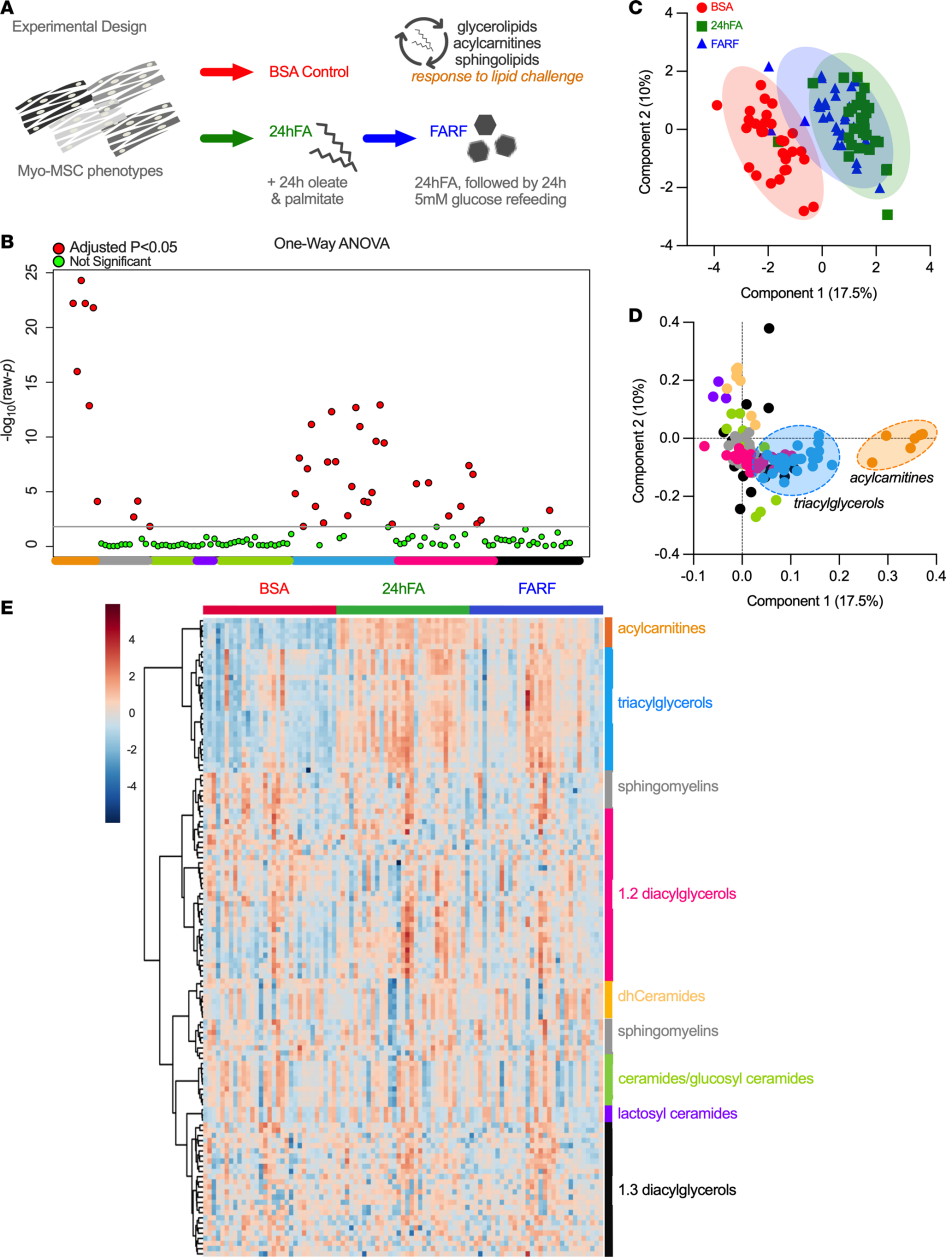
Acylcarnitines, triacylglycerols, and 1,2-diacylglycerols robustly change in response to lipid challenge in all infants. (**A**) Following myogenic induction, we treated cells with either BSA control, 24hFA, or FARF. Cluster 1, *n* = 9; Cluster 2, *n* = 9; Cluster 3, *n* = 13. (**B**) Manhattan plot shows results from 1-way ANOVA with Fisher’s correction for multiple testing. (**C**) Partial least-squares discriminant analysis (PLS-DA) shows that 24hFA shifts metabolism, with partial return to BSA levels following the FARF condition. (**D**) PLS-DA loadings plots shows component 1 accounts for 17% of the variance and is driven by changes in acylcarnitines, triacylglycerols, and 1,2-diacylglycerols. (**E**) A heatmap generated in Metaboanalyst shows conditions for all lipid species.

To test the second part of our hypothesis that MSC lipid metabolism phenotype is defined by the ability to respond to a lipid challenge, we exposed cells to 24 hours of excess oleate/palmitate lipid mix (24hFA) and 24hFA followed by a return to regular 5 mM glucose media refeeding (FARF) (Figure 3A). Among all MSC cell lines, we quantified lipid species for glycerolipids, acylcarnitines (ACs), and bioactive sphingolipids to comprehensively assess response to metabolic challenge. In all participants, 40 species changed in response to lipid challenge, mainly TAGs, DAGs, and ACs (Figure 3B; ANOVA results in Supplemental Table 4). PLS-DA shows lipid species shifted with 24hFA relative to BSA, and partially shifted back toward BSA with the FARF condition (Figure 3C), where component 1 accounted for 17.5% of the variance, mainly driven by changes in ACs and TAGs (Figure 3D). The heatmap reveals consistent patterns within lipid species, with AC, TAG, and 1,2-DAG (Figure 3E), as well as several sphingomyelins (SMs) (23:4, 14:0, 24:2, and 20:1; Supplemental Table 4) being the most robustly changed across all MSCs.

### MSC Cluster 2 has the most robust response to lipid challenge.

We then examined each cluster by PLS-DA to explore differences in response to lipid challenge by cluster (Supplemental Tables 5–7 and Supplemental Figure 2; lipid species by maternal BMI in Supplemental Table 8). Cluster 2 appears to have the most robust change overall, with minimal overlap of the 95% confidence band in the shift from BSA to 24hFA (Supplemental Figure 2). Of note, the BSA condition normalized differences in the sum of TAG species from untreated myogenesis (Figure 1E and Supplemental Figure 3), with the most robust change in Cluster 2. By repeated-measures ANOVA, all clusters exhibited a change in the sum of total lipids in response to 24hFA (*P*_Condition_ < 0.001; Supplemental Figure 4).

**Figure 4:**
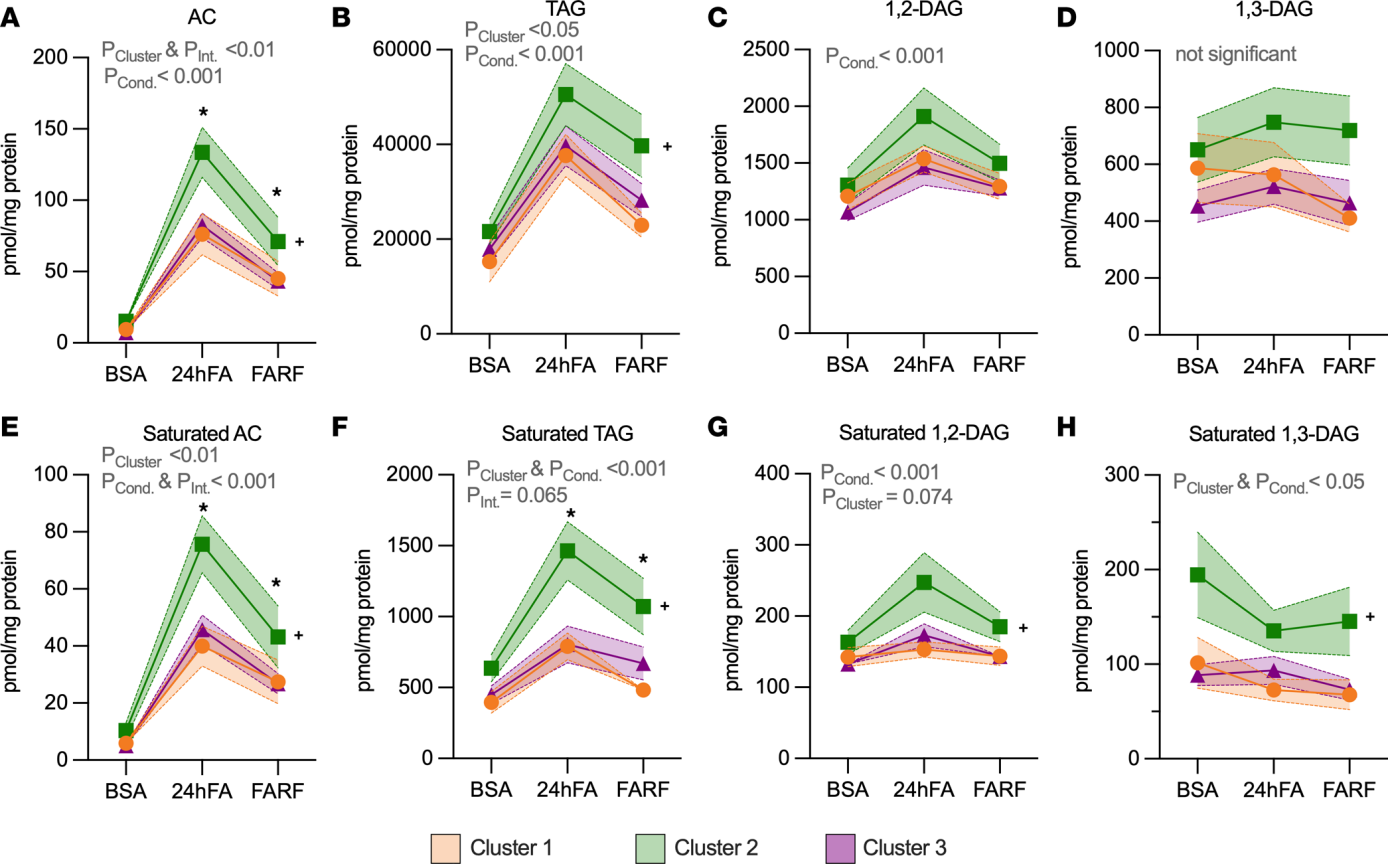
MSC Cluster 2 has the most robust response to lipid challenge for AC, TAG, and DAG species. We calculated the sum of each lipid class in the lipid challenge conditions (BSA, 24hFA, FARF) for all 3 clusters (Cluster 1, *n* = 9; Cluster 2, *n* = 9; Cluster 3, *n* = 13). Data are the changes in the sum of all acylcarnitines (ACs) (**A**), sum of all triacylglycerols (TAGs) (**B**), sum of all 1,2-diacylglycerols (DAGs) (**C**), sum of all 1,3-DAGs (**D**), and the saturated subspecies of these lipids (**E**–**H**). We analyzed data using the GEE. *P < 0.05, Cluster 2 different from Clusters 1 and 3 in the designated condition. **^+^***P* < 0.05, Cluster 2 different from Clusters 1 and 3 across conditions. *P* values listed in the figure are for main effects of the analysis (e.g., effect of cluster, interaction), *P* values listed in the legend, indicated by symbols in the panels, refer to the post hoc tests (least-squares means). Data are mean ± SEM. Cond., effect of condition; Int., effect of interaction.

**Figure 5:**
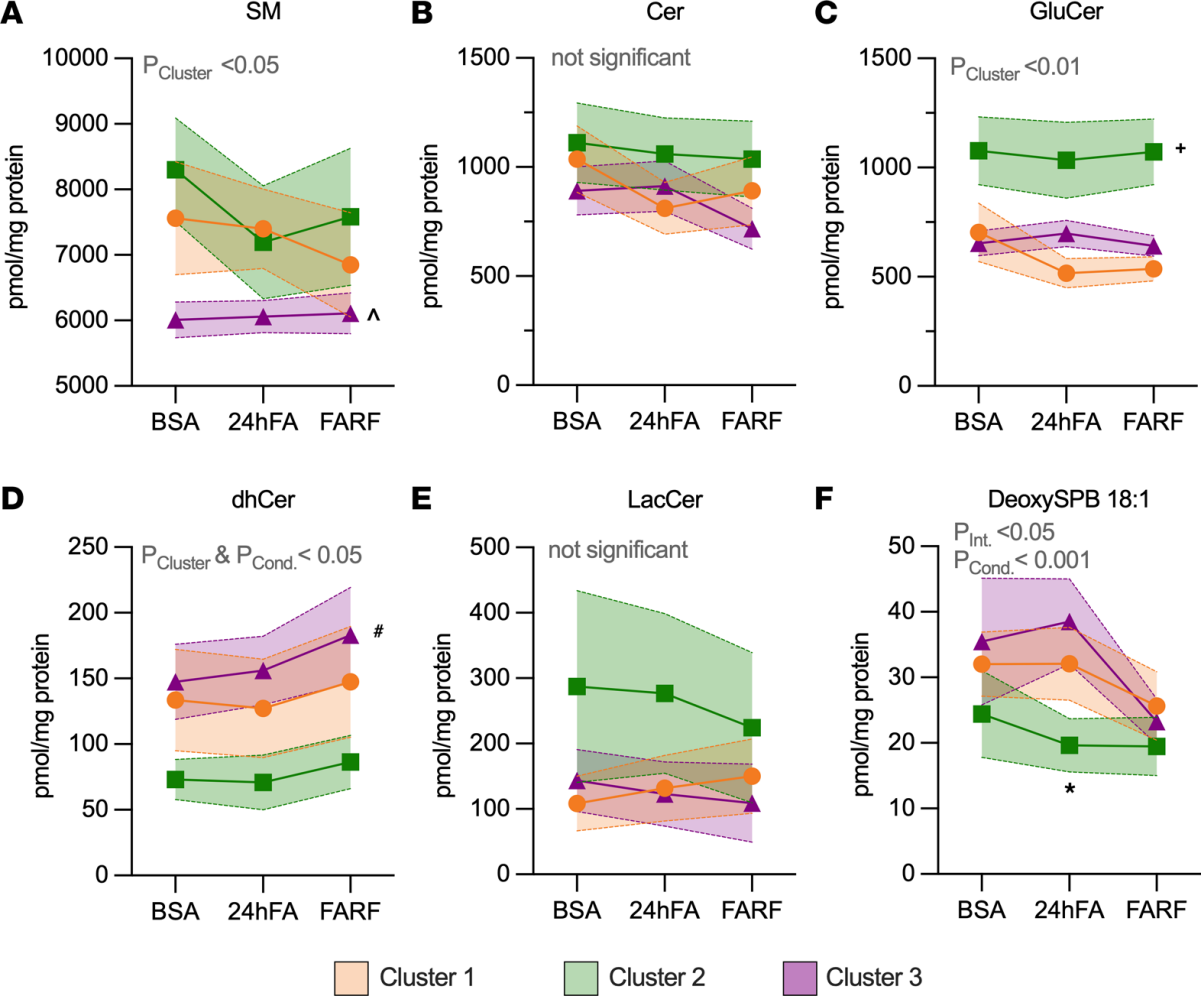
MSC clusters differ in sphingolipid response to lipid challenge. We calculated the sum of each ceramide or sphingomyelin class in the lipid challenge conditions (BSA, 24hFA, FARF) for all 3 clusters (Cluster 1, *n* = 9; Cluster 2, *n* = 9; Cluster 3, *n* = 13). Data are the change in sum of sphingomyelins (SM) (**A**), sum of ceramides (Cer) (**B**), sum of glucosylceramides (GluCer) (**C**), sum of dihydroceramides (dhCer) (**D**), sum of lactosyl ceramides (LacCer) (**E**), and deoxysphingosine (DeoxySPB) 18:1 (**F**). We analyzed data using the GEE. ^*P* < 0.05, Cluster 3 different from Clusters 1 and 2 across conditions. ^+^*P* < 0.05, Cluster 2 different from Clusters 1 and 3 across conditions. ^#^*P* < 0.05, Cluster 3 different from Cluster 2 across conditions. *P < 0.05, Cluster 2 different from Clusters 1 and 3 in the designated condition. *P* values listed in the figure are for main effects of the analysis (e.g., effect of cluster, interaction), *P* values listed in the legend, indicated by symbols in the panels, refer to the post hoc tests (least-squares means). Data are mean ± SEM. Cond., effect of condition; Int., effect of interaction.

Repeated-measures modeling confirmed robust lipidomic shifts in response to 24hFA (*P*_Condition_ < 0.001; Figure 4, A–C), with Cluster 2 demonstrating the greatest change in major lipid classes AC and TAG compared with Clusters 1 and 3 (*P*_Cluster_ < 0.05, *P*_Interaction_ < 0.001; Figure 4, A and B). We interrogated saturated lipids separately and observed similar pattens for saturated AC and TAG (*P*_Interaction_ < 0.01; Figure 4, E and F). Although 1,3-DAG did not change in response to lipid challenge (Figure 4D), saturated 1,3-DAG decreased with 24hFA (*P*_Condition_ < 0.05; Figure 4H), and Cluster 2 exhibited elevated saturated 1,2-DAG and 1,3-DAG across all conditions (*P*_Cluster_ < 0.01; Figure 4, G and H). Given that total lipids were also elevated in Cluster 2 relative to other groups, we adjusted for total lipid content and only TAG remained higher in Cluster 2 compared with Clusters 1 and 3 (*P*_Cluster_ < 0.05; Supplemental Figure 5). Repeated-measures models for the sum of sphingolipid species are shown in Figure 5. SMs were highest in Clusters 1 and 2 relative to Cluster 3 across all experiments (*P*_Cluster_ < 0.05; Figure 5A). Although ceramides (Cer) and lactosyl ceramides (LacCer) did not differ between groups or experiments (Figure 5, B and E), glucosyl ceramides (GluCer) were highest in Cluster 2 and dihydroceramide (dhCer) was highest in Cluster 3, relative to the other clusters (*P*_Cluster_ < 0.05; Figure 5, C and D). Although Cluster 2 responded differently to the lipid stressors with respect to deoxysphingosine (deoxySPB) 18:1 (*P*_Interaction_ < 0.05; Figure 5F), the patterns are less clear.

Overall, Cluster 2 demonstrated the most robust shifts in lipid species with lipid challenge, mainly in ACs and TAGs. There were more subtle differences in other species, including lower SMs in Cluster 3 relative to Clusters 1 and 2. However, these lipidomic measurements represent a snapshot of the cell, albeit under various metabolic conditions, and do not quantify metabolic rate or lipid flux. Therefore, we took steps to quantify nutrient sensing and metabolic flux through AMPK phosphorylation and direct measurement of FAO.

### AMPK activity and FAO begin to distinguish lipid metabolism phenotype of Clusters 1 and 3.

**Figure 6:**
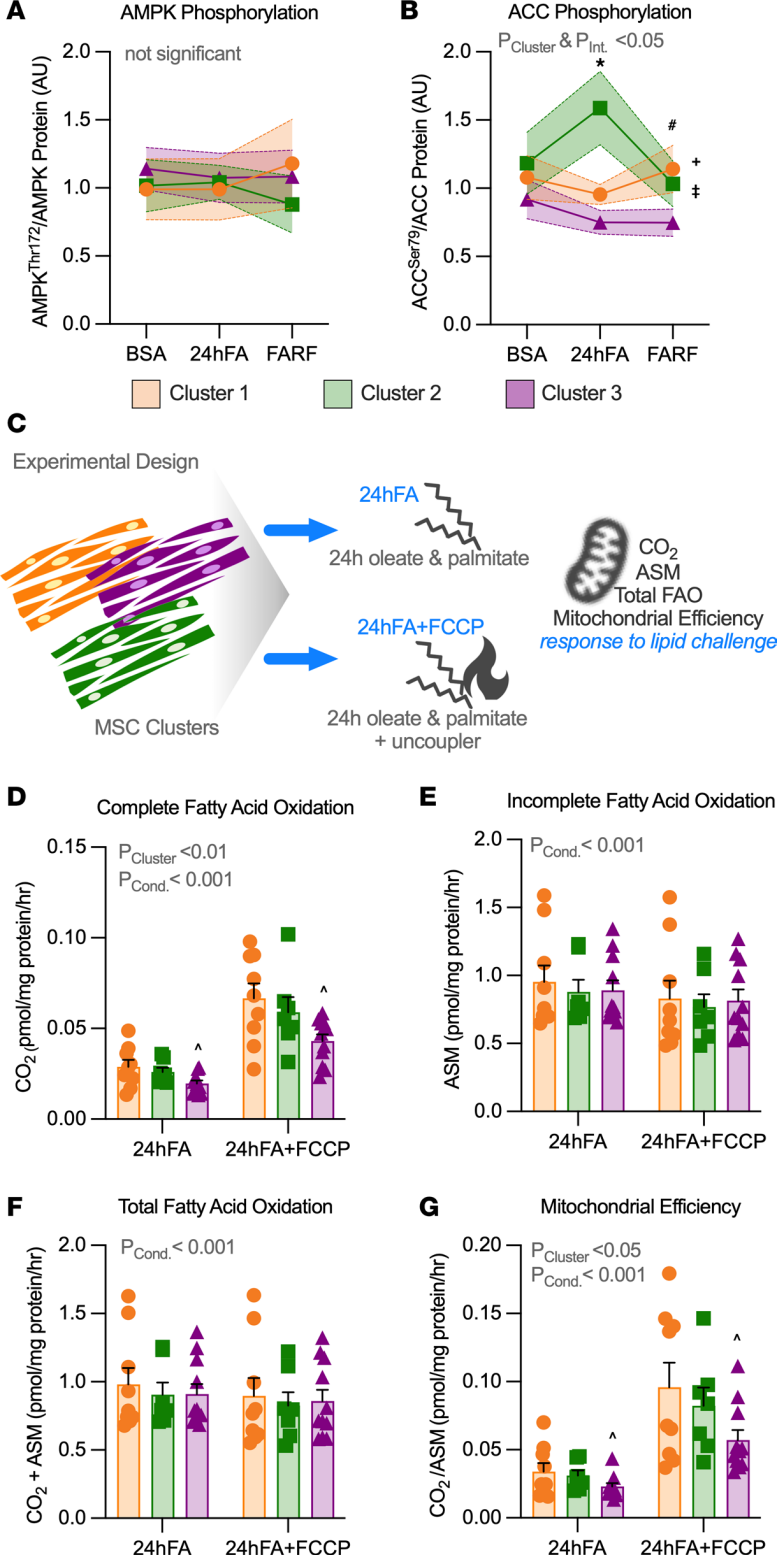
AMPK activity and FAO begin to distinguish lipid metabolism phenotype of Clusters 1 and 3. (**A** and **B**) We measured protein phosphorylation of AMPK and its substrate ACC, an index of AMPK activity, in response to the lipid challenge conditions (BSA, 24hFA, FARF) for all 3 clusters. Data are changes in phosphorylated/total AMPK (AMPK^Thr172^/AMPK) (**A**) and ACC (ACC^Ser79^/ACC) (**B**). Cluster 1, *n* = 9; Cluster 2, *n* = 8; Cluster 3, *n* = 11. (**C**) To support interpretation of the shift in lipid species during lipid challenge, fatty acid oxidation (FAO) was measured in response to the lipid challenge condition (24hFA) and additionally in the context of a mitochondrial uncoupler to allow for maximal lipid oxidation (24hFA+FCCP) for all 3 clusters. Cluster 1, *n* = 9; Cluster 2, *n* = 7; Cluster 3, *n* = 11. Data are complete FAO (**D**), acid soluble metabolites (ASM), an index of incomplete FAO (**E**), total FAO (**F**), and mitochondrial FAO efficiency (**G**; ratio of complete FAO/incomplete FAO, with higher values indicating greater efficiency). We analyzed data using the GEE. ^Cluster 3 different from Clusters 1 and 2 across conditions (*P* < 0.05), ^+^Cluster 2 different from Clusters 1 and 3 across conditions (*P* < 0.05), ^‡^Cluster 1 trended different from Clusters 3 across conditions (*P* = 0.10), *Cluster 2 different from Clusters 1 and 3 in the 24hFA condition (*P* < 0.05), ^#^Cluster 1 trended different from Cluster 3 in the FARF condition (*P* = 0.07). *P* values listed in the figure are for main effects of the analysis (e.g., effect of cluster, interaction), *P* values listed in the legend, indicated by symbols in the panels, refer to the post hoc tests (least-squares means). Data are mean ± SEM.

We examined nutrient sensing in response to lipid challenge through AMPK phosphorylation (AMPK^Thr172^) and acetyl-CoA carboxylase (ACC) phosphorylation. The ratio of phosphorylated/total AMPK (AMPK^Thr172^/AMPK) did not differ by cluster or in response to the lipid challenge (Figure 6A). However, AMPK activity, estimated by phosphorylation of its substrate ACC (ACC^Ser79^/ACC), robustly increased with 24hFA in Cluster 2, but not in Cluster 1 or 3 (*P* = 0.027; Figure 6B). Across all conditions, Cluster 2 exhibited higher ACC^Ser79^/ACC relative to Cluster 3 (*P* = 0.008), and ACC^Ser79^/ACC trended higher in Cluster 1 relative to Cluster 3 (*P* = 0.10). In FARF, Cluster 1 tended to maintain higher ACC^Ser79^/ACC compared with Cluster 3 (*P* = 0.068).

To examine whether this translated to differences in FAO, we designed an experiment to assess FAO under 24hFA lipid stress in “resting” cells, and cells with increased metabolic demand using the chemical uncoupler carbonylcyanide-*p*-trifluoromethoxyphenylhydrazone (FCCP, [24hFA+FCCP]) to account for intrinsic differences in metabolic rate among the MSC cell lines (Figure 6C). BSA and FARF conditions were not included in this experiment because the FAO assessment itself exposes the cells to fatty acids, prohibiting a fatty acid–free measurement comparable to the lipidomic measurements. Complete oxidation of fatty acids to CO_2_ was lowest in Cluster 3 in both 24hFA and maximally stimulated FAO (24hFA+FCCP) conditions, relative to Clusters 1 and 2 (*P*_Cluster_ < 0.01; Figure 6D). Incomplete FAO (acid-soluble metabolites, ASM) and total FAO did not differ by cluster (Figure 6, E and F). However, mitochondrial efficiency for FAO (CO_2_/ASM) was highest in Clusters 1 and 2, relative to Cluster 3 under both 24hFA and 24hFA+FCCP conditions (*P*_Cluster_ < 0.05; Figure 6G).

## Discussion

This study was designed to comprehensively characterize MSC lipid metabolism phenotypes in obesity- and non–obesity-exposed infants to more finely distinguish potential maternal effectors affecting offspring obesity risk. Accordingly, we performed machine learning techniques to cluster MSCs based on storage of glycerolipid species, which represents cumulative lipid handling over the course of myogenesis, revealing 3 distinct lipid-derived MSC clusters. We then assessed how these phenotypes responded to metabolic challenge, a key index of cellular metabolic health, with respect to glycerolipids, ACs, and bioactive sphingolipids. Lastly, we observed that these MSC clusters strongly associate with the maternal metabolic milieu, independent of maternal ppBMI, and prospectively associate with child adiposity through 4–6 years. Specifically, the composite maternal milieu score was nearly 60% higher in Cluster 3 relative to Clusters 1 and 2, and children from Cluster 3 had 10% higher adiposity than children in Clusters 1 and 2 at 4–6 years.

All experiments were performed in MSC-derived myotubes, and therefore are a model of metabolically active skeletal muscle tissue. As such, the ability to respond to prevailing metabolic demands, particularly varied loads of nutrient substrates, is requisite for these cells/tissues. In these data, we utilized quantitative lipidomic measurements to observe complementary measurements of cellular lipid handling and response to metabolic demand. First, we measured the cumulative lipid stores over the course of 21 days of myogenesis, and then we measured changes in lipid species in response to 24FA lipid challenge studies. During myogenesis and in the 24FA experiments, lipid uptake may outpace the capacity for lipid oxidation, spilling over into longer-term lipid storage. Thus, the higher TAG content in Cluster 2 in the initial clustering analysis may be indicative of more favorable lipid storage, as opposed to bioactive lipid intermediates such as DAG or Cer (15). This may explain why TAGs were the most important features in the initial clustering. Although there appears to be an increase in 1,2-DAG in all MSC clusters, when total lipids are accounted for there is a proportional decrease in DAGs in response to the 24hFA lipid challenge. Therefore, even though Cluster 2 exhibits the highest amount of total lipids and TAG, the ability to shift excess lipids toward β-oxidation and biologically neutral TAG storage likely contributes to the favorable metabolic profile exhibited in our complementary measurements of AMPK activation and FAO. This is consistent with myocyte studies from healthy insulin-sensitive adults, where lipids are appropriately mobilized and lipid oxidation is higher during 24-hour (16) or 3-day (17) lipid challenge relative to adults with established obesity. Moreover, our prior report demonstrated that MSCs with the greatest TAG content had the highest insulin action (10). Similar to the “athletes’ paradox” (11), it appears that total lipids alone do not define MSC phenotypes, but rather the types of lipids and response to metabolic challenge.

Although all MSC cell lines demonstrate shifts in ACs, TAGs, and DAGs in response to lipid challenge, the most robust changes were observed in ACs and TAGs in MSC Cluster 2. While medium- and short-chain fatty acids can freely enter the mitochondria, AC formation is necessary for movement of long-chain fatty acids (such as oleate and palmitate used here) into mitochondria for β-oxidation (18). Thus, AC increases may indicate oxidation of the excess lipids (i.e., metabolic flexibility; ref. 19). This is corroborated by the robust Cluster 2 increase in AMPK activity (ACC phosphorylation) with 24hFA. AMPK responds to the cellular energetic state (ADP, ATP) and stimulates FAO via ACC inhibition (20). However, given that Clusters 1 and 3 exhibit increases in AC with 24hFA in the absence of increased ACC phosphorylation, other factors likely also regulate lipid metabolism. It is possible that lipid availability, basal cellular metabolic rate, or energetic demand for lipids may impact these results. For example, energetic activation of AMPK could increase AMPK substrate phosphorylation (ACC), even without substantial changes in AMPK phosphorylation at Thr172. Given expected differences in lipid content among MSC clusters, which appear to be linked to different responses to the lipid challenge, comparison of endogenous versus exogenous substrate oxidation under similar conditions may further delineate MSC metabolic phenotypes linked to childhood adiposity.

To address potential limitations in metabolism due to lipid availability or energetic demand, we directly measured FAO in the 24hFA condition and with the chemical uncoupler FCCP added, which increases metabolic rate and FAO. In these experiments, we did not observe differences in total FAO, but Clusters 1 and 2 show greater oxidation of fatty acids to CO_2_ (complete FAO) compared with Cluster 3, which translates to greater mitochondrial efficiency and supports the metabolic health of both Clusters 1 and 2 over Cluster 3 through factors beyond large shifts in AC. We suspect Cluster 3 may have persistent limitations in metabolic activity, given that Cluster 3 demonstrated the lowest AMPK activity across all metabolic conditions and exhibited intrinsic deficits in complete FAO in response to 24-hour lipid challenge, even when we stimulated maximal flux through the electron transport chain (via FCCP) to rule out energetic demand as a contributor. Persistent limitations in Cluster 3 are further supported by the dhCer and SM measurements, where Cluster 3 exhibits the highest levels of dhCer, but the lowest levels of SM, compared with Clusters 1 and 2. dhCer are bioactive lipids and a risk marker for type 2 diabetes (21), while SMs are important for membrane structure and transport. Although SMs are also the pool of lipids from which Cer are formed, if more are shunted to membrane structure, higher SM in Clusters 1 and 2 may be protective against accumulation of bioactive ceramides (22), which would support cellular metabolic health and decrease risk for insulin resistance and type 2 diabetes (reviewed in ref. 15). We note that, even though Cluster 2 had higher levels of most lipid species relative to Clusters 1 and 3, Cer levels were not elevated, which may indicate a buffering effect. Our lipidomic measurement did not include all forms of membrane lipids, which are of interest in metabolic activity (23), and these bear inclusion in future studies.

To determine how fetal exposures may broadly contribute to the observed MSC phenotypes, we calculated a cumulative index of maternal metabolic milieu across pregnancy, which corresponded to MSC cluster. In fact, the maternal stress score was nearly 60% higher in Cluster 3 relative to Clusters 1 and 2. Among the individual components of the maternal stress score, we note that maternal lipids (i.e., triglycerides and FFAs) and TNF-α most strongly predict MSC cluster, supporting the importance of studying maternal factors beyond glucose and insulin in the context of the developmental origins of obesity (24). Consistent with our findings, multiple maternal fuels during pregnancy have been related to child health outcomes (i.e., neonatal %FM) in the larger Healthy Start parent cohort (25). Most recently, we demonstrated in nearly 600 infants that cord blood DNA methylation of lipid metabolism and immune function genes was most robustly associated with maternal triglycerides rather than glucose, insulin, or BMI. Moreover, DNA methylation of these genes predicted childhood adiposity and mediated the relationship between maternal triglycerides and child adiposity (26). Work from other groups has also highlighted maternal lipids (27–29) and markers of inflammation (30) as key predictors of child body composition. Importantly, the MSC clusters also prospectively associate with child adiposity whereby small neonatal differences emerged into larger early childhood differences, such that children from Cluster 3 had 10% higher adiposity than children in Clusters 1 and 2 at 4–6 years. However, these child findings are limited by the smaller sample size at 4–6 years (*n* = 13), and bear repeating in a larger sample. This pattern of increasing impact of fetal exposure over age has been shown in previous studies (31, 32), where the association between maternal BMI and child obesity risk is greater as children grow older and is independent of birthweight. This highlights the promise of the MSC model for identifying cellular biomarkers that predict offspring at risk for obesity development, even among children with normal birthweight and birth adiposity measurements, and moreover, from children born to normal-weight pregnancies. Importantly, these associations between MSC phenotypes and maternal and child features were independent of maternal ppBMI, which supports a causal role of maternal milieu over maternal body size, informing potential targets for pregnancy intervention studies. We note that our sample was enriched for maternal obesity; as blood lipids and other metabolic and proinflammatory factors are more likely to be elevated in this population, this enriched sampling design increases the variance of our predictor variables, thus increasing efficiency and power to detect linear effects in our smaller experimental studies (33, 34).

In summary, this work defines what we believe is a novel MSC lipid phenotype, largely characterized by TAG stores in MSC-derived myocytes, suggesting the initial lipid storage and ability to mobilize lipids may play a protective role by maintaining cellular metabolic health. SMs are another potentially important lipid species highlighted in this work, with lower SMs distinguishing the cluster associated with highest maternal stress exposure and highest offspring adiposity compared with the other 2 clusters. Our data provide further evidence that MSC metabolic phenotypes may distinguish children at risk for excess adiposity, although further work is needed to confirm this in larger longitudinal samples. Moreover, these phenotypes are predicated by key fetal determinants, the maternal metabolic milieu, independent of maternal BMI. New model systems, such as MSCs, are needed for improved precision outcomes to evaluate the effectiveness of early-life intervention efforts.

## Methods

### Sex as a biological variable

This study uses cells and samples from human participants. Pregnant participants were all female, with both male and female infants meeting inclusion/exclusion criteria included in the offspring measurements.

### Participants

#### Maternal measurements.

The Healthy Start cohort study enrolled 1,410 pregnant women at 16 years of age or older and at less than 23 weeks of gestation, from obstetrics clinics at the University of Colorado Hospital from 2009 to 2014. We excluded women with prior diabetes, premature birth, serious psychiatric illness, or a current multiple pregnancy. We cultured umbilical cord MSCs from convenience samples of 165 infants. For this study, additional inclusion criteria included full-term infant (>37 weeks gestation), mother more than 17 years of age, and no gestational diabetes or preeclampsia diagnosis. Sixteen mothers with obesity met these criteria and were frequency matched with 15 normal-weight women for maternal age, gestational age at delivery, infant sex, and MSC culture time to confluence, as described previously (5). We have previously described MSCs from these same infants (5, 6, 9) and, aside from the selection of those with maternal obesity and matched normal weight, the characteristics of our sample reflect the larger Healthy Start cohort (Supplemental Table 1).

Data collection for Healthy Start was previously described (35). Briefly, we evaluated women at mid and late gestation (~17 and ~27 weeks of gestation) for height, weight, self-reported demographic data, and fasted blood samples to measure glucose, insulin, triglycerides, FFAs, TNF-α, cholesterol, and HDL-cholesterol. A composite maternal metabolic milieu score of all metabolic blood measurements was calculated as a sum of *z* scores for each visit. All values were positively combined, except that HDL was subtracted due to the opposite direction of biologic impact (i.e., greater HDL is metabolically beneficial). We obtained ppBMI through medical record abstraction (84%) or self-report at the first research visit (16%).

#### Infant and child measurements.

At birth, we collected umbilical cord tissue for culture of infant MSCs. We also measured insulin, glucose, and triglycerides from umbilical cord blood. We obtained birth weight from medical records and measured weight, length, and body composition (%FM) by whole body air displacement plethysmography (PEA POD, COSMED, Inc.) 24–48 hours after birth (*n* = 29). At 4–6 months (*n* = 29) and 4–6 years of age (*n* = 13), children returned for additional measurements of body composition (BOD POD).

### MSC isolation and differentiation

We cultured MSCs from fresh umbilical cord tissue explants, as described previously (5). This preparation yields a population of cells that is greater than 98% positive for MSC markers (5). We performed all experiments on cells at passages 3–7. We induced myogenesis as described previously with myogenic induction medium containing 5.5 mM glucose (MIM) (5, 6, 36). In our hands, MSCs express appropriate myogenic markers with in vitro induction (5). Our previous report showed no difference in the percentage of myogenin-containing cells after 7 days of myogenesis, as measured by flow cytometry (5). Here, we made measurements in MSCs with myogenic induction for 21 days (Myo-MSC) or in MSCs with myogenic induction plus lipid challenge conditions, as indicated.

### Lipid challenge experiments

Following 19-day myogenesis, we exposed MSCs to a physiologically relevant lipid challenge to determine metabolic flexibility (Figure 1A). First, we replaced MIM with MIM plus 200 μM oleate/palmitate lipid mix (2:1 ratio, BSA-bound at a molar ratio of 2.5:1) plus 1 mM carnitine for 24 hours (24hFA), as described previously (6, 9). This physiological blend of oleate, palmitate, and carnitine (as opposed to palmitate alone) approximates in vivo fuel metabolism and lipid challenge without inducing cytotoxic stress (37). The molar lipids/BSA ratio of 2.5:1 approximates that in human serum (38). Vehicle control was 24-hour 0.5% BSA in MIM. After 24 hours, we rinsed cells with PBS and returned MSCs to standard MIM for an additional 24 hours (FARF). We harvested cells from all conditions on day 21 of myogenesis. We also collected samples after 21 days of myogenesis with no lipid treatment (Myo Control).

### Protein measurements

We harvested MSCs at indicated time points in ice-cold lysis buffer (CelLytic MT, Sigma-Aldrich) supplemented with protease and phosphatase inhibitors (Sigma-Aldrich). We determined total protein by bicinchoninic acid (BCA) assay. We used Simple Western (JESS, ProteinSimple) to measure total protein and abundance of phosphorylated (AMPK^Thr172^) and total AMPK, and its downstream substrate ACC (ACC^Ser79^), as described previously (6). Antibody specifics and assay conditions are listed in Supplemental Table 2, and chemiluminescent tracings are shown in Supplemental Figure 1.

### Lipidomics

We harvested cell pellets at indicated time points and immediately flash froze them in liquid N_2_. Cells were thawed on ice, resuspended in PBS, and then supplemented with internal standards. Lipids were extracted and analyzed by the Colorado Nutrition Obesity Research Center Molecular and Cellular Analytic Core, as previously described (39). Samples were run on a SCIEX 2000 triple quadrupole mass spectrometer (Applied Biosystems). Lipid species concentration was determined by comparing ratios of unknowns to odd-chain or deuterated internal standards and compared to standard curves run with standards of each lipid species.

### FAO measurements

We assessed day 21 myogenic MSC ^14^C-labeled FAO in 2 conditions: (a) following 24hFA, and (b) following 24hFA with FAO measurements in the presence of 8 μM FCCP (24hFA+FCCP). FCCP uncouples oxidative phosphorylation, effectively increasing metabolism to maintain the mitochondrial membrane proton gradient. This allowed us to rule out differences in metabolic demand as a potential factor contributing to MSC differences in lipid oxidation, as previously described (6, 9). Briefly, on day 21, following 24hFA cells were incubated for 2 hours with the same 24hFA lipid and carnitine lipid mix, spiked with 0.25 μCi/mL [^14^C]-oleate and 0.25 μCi/mL [^14^C]-palmitate (PerkinElmer Life Sciences) (6). FAO was determined by measuring ^14^CO_2_ released from the media after acidification with perchloric acid, in triplicate, and corrected for total protein content. ASMs were measured as an index of incomplete FAO. We calculated total FAO as the sum of ASM plus CO_2_, and mitochondrial efficiency for FAO as the ratio of CO_2_/ASM (greater CO_2_/ASM indicating greater efficiency).

### Statistics

Significance was indicated at an α value of 0.05. We preprocessed the lipidomic data as follows: First, we removed features with greater than 25% missingness across samples due to undetectable or below limit of quantification (BLOQ) values. Next, for features with undetectable or BLOQ values in less than 25% of samples, we replaced missing values with half the minimum value from the existing feature measurement. Last, we removed features more than 3.5 times the upper limit of quantification in more than 25% of samples. In total, we removed 22 features, leaving 124 lipid features analyzed in this study.

We performed initial analyses using Metaboanalyst 5.0 (40) or R Studio 12.0 (https://www.r-project.org). We mean-centered and log- or cube-root-transformed data, where necessary. We performed cluster analysis using K-means clustering (3 groups) with standard settings in Metaboanalyst. We limited the cluster analysis to species of interest based on our a priori hypothesis that lipid metabolic phenotype of the cells is dependent on type and pattern of initial lipid stores: TAGs, and DAGs. In all MSC lines, we performed 1-way analysis of variance (ANOVA) with Fisher’s post hoc test and PLS-DA to determine effect of lipid challenge conditions and important features changed with lipid exposure (24hFA) and glucose-only refeeding (FARF).

For repeated measures, we modeled cluster comparisons using a population-averaged generalized estimating equation (GEE) in SAS software version 9.4 (https://www.sas.com). GEE modeling accounts for intraindividual correlation between repeated measures across time/condition, tests for interaction between cluster and experimental time/condition, and allows for inclusion of participants with missing data across time/condition. A significant cluster × condition interaction indicates that the effect of cluster varies by condition, and the magnitude is even stronger in the specified conditions. For significant effects of cluster or significant effect of cluster × condition, we performed post hoc analysis by least-squares means (LS-means). For association models with maternal or child characteristics, we selected a priori covariates (maternal age, parity, ppBMI, infant sex, and child age at measure or maternal gestational age at measurement). To support interpretation of the maternal metabolic milieu score, we calculated the percentage difference between MSC clusters by log transforming the response variable and applying the formula 100(*e*^βc^ – 1)% to the estimated differences (41). Type 3 analysis using Wald’s test was performed when a priori covariates were included in the model.

### Study approval

This study used umbilical cord tissue samples and data collected as part of the Healthy Start study (ClinicalTrials.gov NCT02273297). The study was approved by the Colorado Multiple Institutional Review Board at the University of Colorado Hospital. At enrollment, written, informed consent was obtained from all participants before participation in the study.

### Data availability

All lipidomics data and all underlying data used to generate graphed means for clinical characteristics and individual MSC outcomes are available in the supplemental Supporting Data Values file.

## Author contributions

KEB conceived this project and designed the experiments. AMD, BCB, KEB, and VZ performed the experiments. KEB and LEG analyzed the data and drafted the manuscript. KEB, MEL, and LEG interpreted the results. DD conceptualized and implemented the parent Healthy Start study. All authors edited the manuscript and approved the final version of the manuscript. KEB is the guarantor of this work and, as such, had full access to all the data in the study and takes responsibility for the integrity of the data and the accuracy of the data analysis.

## Supplementary Material

Supplemental data

Supporting data values

## Figures and Tables

**Table 1:**
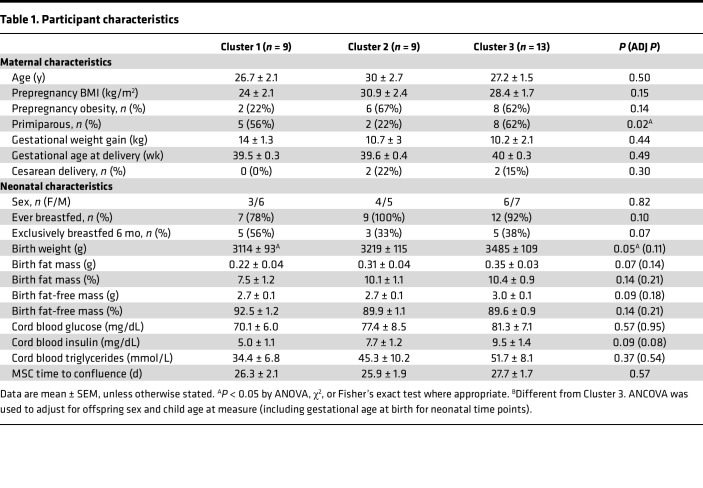
Participant characteristics

## Version 1 09/03/2024

In-Press Preview

## Version 2 10/08/2024

Electronic publication
