# Supplementary material for: Lipidomics of infant mesenchymal stem cells associate with the maternal milieu and child adiposity
Source: JCI Insight. 2024 Sep 3;9(19):e180016. doi: 10.1172/jci.insight.180016 (PMC11466181; doi:10.1172/jci.insight.180016)
Supplement: Supplemental data [file jciinsight-9-180016-s074.pdf]

Supplemental Figures & Tables for:

Title: Lipidomics of infant mesenchymal stem cell associate with the maternal milieu and child adiposity

Authors:
Lauren E. Gyllenhammer[1], Vincent Zaegel[2], Allison M. Duensing[2], Manoel Lixandrao[2], Dana Dabelea[3,4], Bryan C. Bergman[5], and Kristen E. Boyle[2,3]

Affiliations:
[1]University of California, Irvine, School of Medicine, Department of Pediatrics, Irvine, CA USA.
[2]Section of Nutrition, Department of Pediatrics, University of Colorado Anschutz Medical Campus, Aurora, CO USA.
[3]The Lifecourse Epidemiology of Adiposity and Diabetes (LEAD) Center, Aurora, CO USA.
[4]Department of Epidemiology, Colorado School of Public Health, Department of Pediatrics, University of Colorado Anschutz Medical Campus, Aurora, CO USA.
[5]Department of Endocrinology, Metabolism, and Diabetes, University of Colorado Anschutz Medical Campus, Aurora, CO USA.

Corresponding Author:
Kristen E. Boyle, PhD
kristen.boyle@cuanschutz.edu
303-724-5969
12700 East 19th Ave, Mail Stop C-225
Research Complex II
Aurora, CO 80045

Data Availability

All lipidomics data and all underlying data used to generate graphed means for clinical characteristics and individual MSC outcomes are available in the Supporting Data Values file.

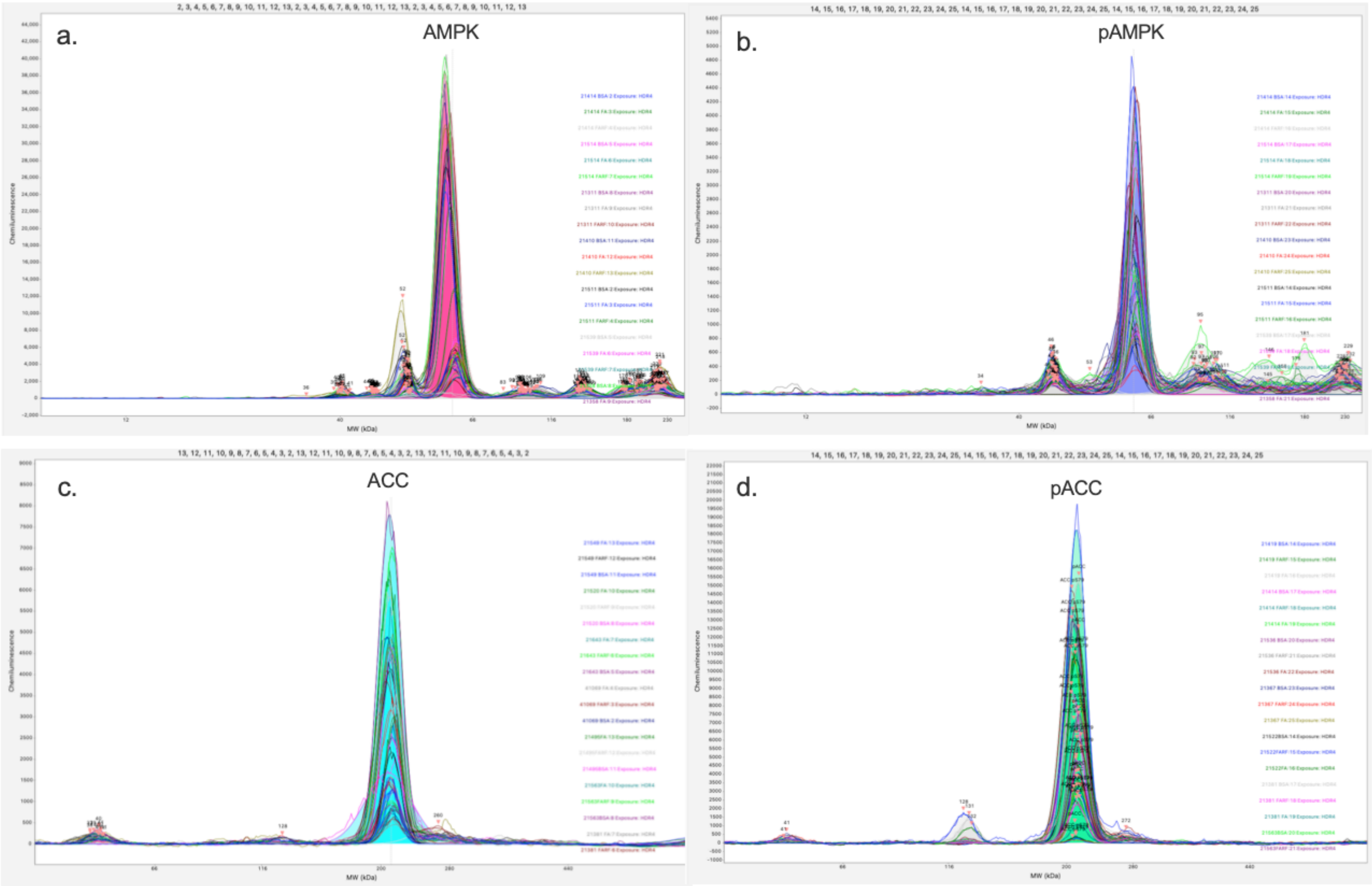

Supplemental Figure 1. Representative and group mean tracings of WES Spectra Plots for AMPK (a), AMPK^{Thr172} (b), ACC (c) and ACC^{Ser79} (d).

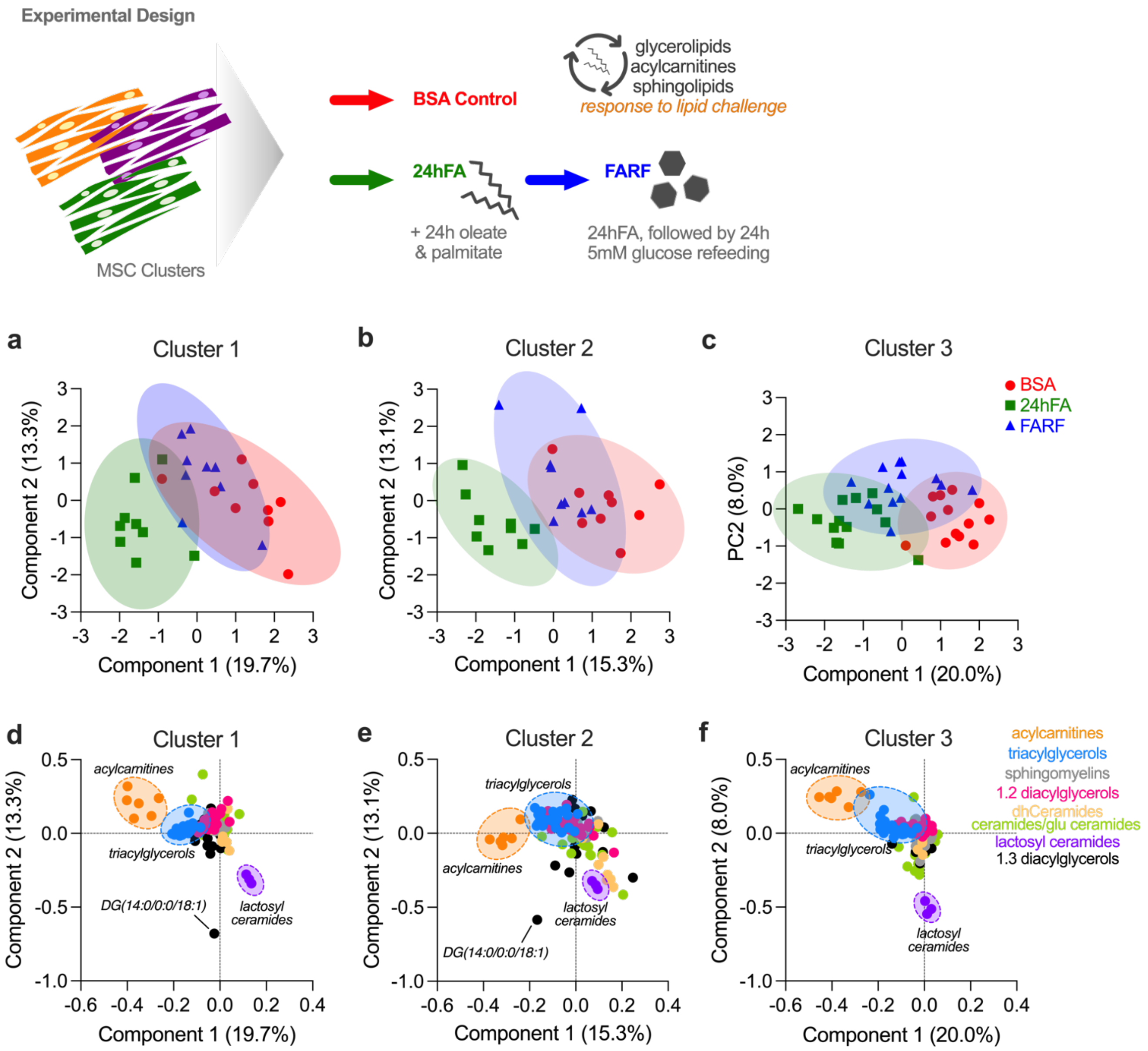

Supplemental Figure 2. MSC clusters lipid species differ in response to lipid challenge. We performed partial least-squares discriminant analysis for all lipid species in the three lipid challenge conditions (BSA, 24hFA, and FARF). For all Clusters, Components 1 and 2 explained ~30-35% of the variance and shifts in acylcarnitine (AC) largely influenced Component 1 (a-c). For Cluster 2, AC, dihydroceramide (dhCer), and lactosyl ceramide (LacCer) species also influenced Component 2, (b). For Cluster 1, LacCer and glucosyl ceramide (GluCer) species influenced Component 2 (a), while dhCer influenced PC2 in Cluster 3 (c). There was no discernible pattern for 1,3-DAG or sphingomyelins (SM) influence on the PLS-DA components, though Clusters 1 and 2 showed influence of C14:0/18:1 1,3-DAG separate from other 1,3-DAG species.

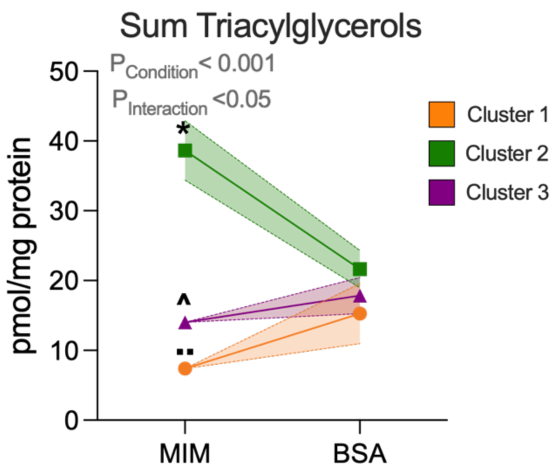

Supplemental Figure 3. Bovine serum albumin (BSA) condition induces normalization of stored triacylglycerols.

Changes in the sum of triacylglycerols (TAG) in response to the lipid carrier BSA "control" condition (BSA) relative to the Myo-induction media (MIM) condition for all three clusters. We analyzed data using generalized estimating equation modeling.

*Cluster 2 different from Clusters 1 & 3, in the designated condition. ¨ Cluster 1 different from Clusters 2 & 3, in the designated condition. ^Cluster 3 different from Clusters 1 & 2, in the designated condition. P<0.05, data are mean ± SEM.

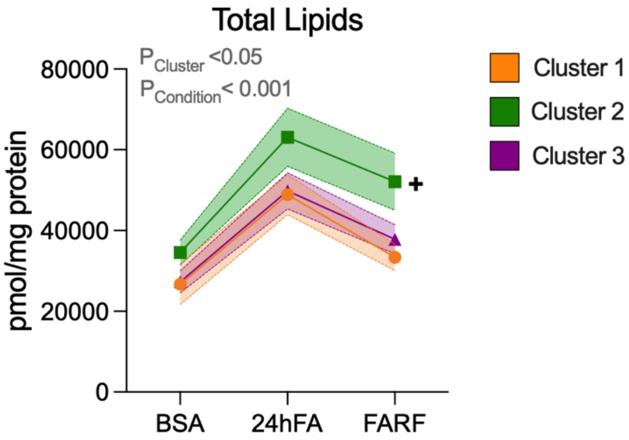

Supplemental Figure 4. MSC clusters differ in total lipid response to lipid challenge

Total measured lipids changed in response to the lipid challenge conditions (BSA, 24hFA, FA+RF) and by MSC Cluster. We analyzed data using generalized estimating equation modeling.

+Cluster 2 different from Clusters 1 & 3 across conditions. P<0.05, data are mean ± SEM.

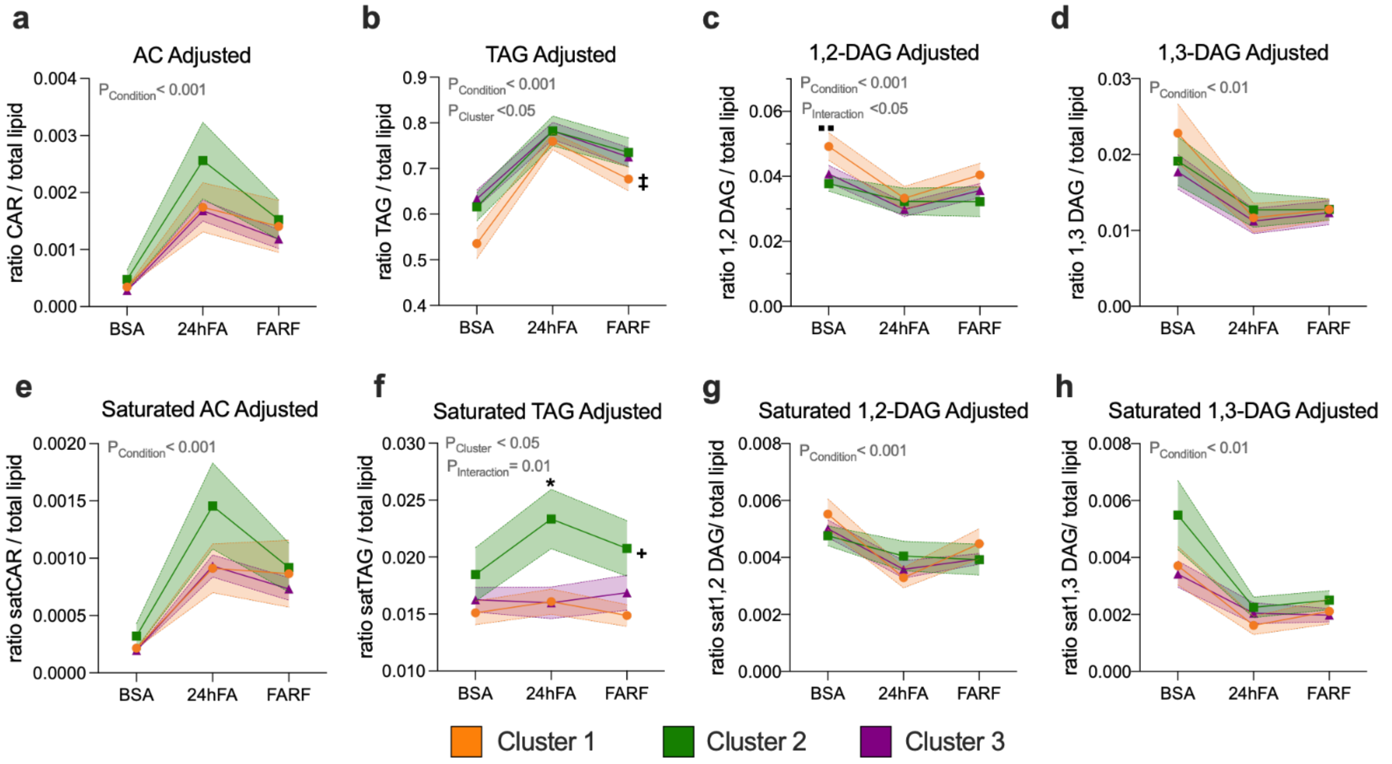

Supplemental Figure 5. MSC clusters differ in CAR, TAG, and DAG response to lipid challenge after taking into account total lipids. We calculated the sum of each lipid class across the lipid challenge experiment (BSA, 24hFA, FARF) for all three clusters, and adjusted each lipid species for total lipids. Data are the changes in the sum of all acylcarnitines (CAR) adjusted for total lipids (a), sum of all triacylglycerols (TAG) adjusted for total lipids (b), sum of all 1,2-Diacylglycerols (DAG) adjusted for total lipids (c), sum of all 1,3-DAG adjusted for total lipids (d), and the saturated subspecies of these lipids (e-h). We analyzed data using generalized estimating equation modeling.

*Cluster 2 different from Clusters 1 & 3, in the designated condition. +Cluster 2 different from Clusters 1 & 3 across conditions. ‡Cluster 1 different from Clusters 2 & 3 across conditions, ¨Cluster 1 different from Clusters 2 & 3 in the designated condition. $P<0.05$, data are mean ± SEM.

List of Supplementary Tables

Table S1. Participant characteristics by maternal obesity status and relative to full Healthy Start Cohort
Table S2. Antibodies for simple western
Table S3. Markers of myogenic differentiation and characteristics by Cluster
Table S4. Individual Lipid Species with ANOVA P<0.05 between lipid challenge conditions, all infants
Table S5. Individual Lipid Species with ANOVA P<0.05 between lipid challenge conditions, Cluster 1
Table S6. Individual Lipid Species with ANOVA P<0.05 between lipid challenge conditions, Cluster 2
Table S7. Individual Lipid Species with ANOVA P<0.05 between lipid challenge conditions, Cluster 3
Table S8. Individual Lipid Species across lipid challenge conditions, with t-test between normal weight or obese pregnancies conditions, all infants

Table S1. Participant characteristics by maternal obesity status and relative to full Healthy Start Cohort

	Normal Weight (n = 15)	Obese (n = 16)	All (n = 31)	P Value (ttest NW vs. Ob)	Larger Healthy Start Cohort		
					Statistic	*N*	*Ref.*
**Maternal Characteristics**							
Age (y)	28.3 ± 1.4	27.1 ± 1.7	27.6 ± 1.1	0.5919	27.8 ± 6.2	*1,325*	From: doi:10.1001/jamanetworkopen.2023.7030
Primiparous, *n* (%)	8 (53%)	7 (44%)	15 (48%)	0.5936	689 (52%)	*1,325*	From: doi:10.1001/jamanetworkopen.2023.7030
Cesarean delivery, n (%)	2 (13%)	2 (13%)	4 (13%)	0.9449	226 (21%)	*1,102*	From: https://doi.org/10.1016/j.jpeds.2017.01.014
Pre-pregnancy BMI (kg/m^2)	21.2 ± 0.3	33.5 ± 0.8	27.5 ± 1.2	<.0001*	25.7 ± 6.1	*1,325*	From: doi:10.1001/jamanetworkopen.2023.7030
Gestational weight gain (kg)	13.6 ± 0.8	9.5 ± 2.2	11.4 ± 1.2	0.0967	13.6 ± 6.5	*1,102*	From: https://doi.org/10.1016/j.jpeds.2017.01.014
Gestational age at delivery (wk)	39.8 ± 0.3	39.6 ± 0.3	39.7 ± 0.2	0.5940	39.4 ± 1.6	*1,102*	From: https://doi.org/10.1016/j.jpeds.2017.01.014
Insulin (nIU/mL)	12.8 ± 2.3	15.4 ± 1.6	14.1 ± 1.4	0.3643	17.9 ± 16.8	*1,325*	From: doi:10.1001/jamanetworkopen.2023.7030
Glucose (mg/dL)	77.6 ± 2.8	79.9 ± 2.0	78.8 ± 1.7	0.5022	78.0 ± 8.5	*1,325*	From: doi:10.1001/jamanetworkopen.2023.7030
HDL-C (mg/dL)	65.5 ± 4.4	57.9 ± 3.0	61.5 ± 2.7	0.1572	63.4 ± 13.4	*1,325*	From: doi:10.1001/jamanetworkopen.2023.7030
Triglycerides (mg/dL)	159.1 ± 12.4	166.3 ± 10.8	162.8 ± 8.1	0.6644	163.2 ± 62.0	*1,325*	From: doi:10.1001/jamanetworkopen.2023.7030
Free Fatty Acids (mg/dL)	377.5 ± 28.6	438.3 ± 33.2	408.9 ± 22.3	0.1786	372.4 ± 147.1	*1,325*	From: doi:10.1001/jamanetworkopen.2023.7030
**Neonatal Characteristics**							
Sex, *n* (f/m)	7/8	6/10	13/18	0.6052	536/566	*1,102*	From: https://doi.org/10.1016/j.jpeds.2017.01.014
Ever breastfed, *n* (%)	13 (87%)	15 (94%)	28 (90%)	0.5996			
Exclusive breastfed 6 mo, *n* (%)	8 (53%)	5 (31%)	13 (42%)	0.2131	393 (42%)	*1,102*	From: https://doi.org/10.1016/j.jpeds.2017.01.014
Birth weight (g)	3287 ± 101	3312 ± 91	3300 ± 68	0.8564	3240 ± 500	*1,102*	From: https://doi.org/10.1016/j.jpeds.2017.01.014
Birth fat mass (kg)	0.26 ± 0.03	0.34 ± 0.03	0.30 ± 0.02	0.0733	0.30 ± 0.15	*1,102*	From: https://doi.org/10.1016/j.jpeds.2017.01.014
Birth fat mass (%)	8.3 ± 0.9	10.8 ± 0.7	9.5 ± 0.6	0.0494*	9.2 ± 3.9	*1,102*	From: https://doi.org/10.1016/j.jpeds.2017.01.014
Birth fat free mass (kg)	2.9 ± 0.1	2.8 ± 0.1	2.8 ± 0.1	0.8042	2.84 ± 0.35	*1,102*	From: https://doi.org/10.1016/j.jpeds.2017.01.014
Birth fat free mass (%)	91.7 ± 0.9	89.2 ± 0.7	90.5 ± 0.6	0.0494*			
Cord blood glucose (mg/dL)	76 ± 6.5	77.8 ± 5.7	77.1 ± 4.2	0.8391	79 (70–90)[#]	*812*	From: doi: 10.1097/EE9.0000000000000203
Cord blood insulin (μIU/ml)	6.0 ± 0.8	9.2 ± 1.2	7.9 ± 0.8	0.0576	7 (5–11)[#]	*812*	From: doi: 10.1097/EE9.0000000000000203
Cord blood triglycerides (mg/dL)	41.5 ± 8.1	47.5 ± 6.4	45.1 ± 5	0.5678	39 (29–55)[#]	*812*	From: doi: 10.1097/EE9.0000000000000203
MSC time to confluence (d)	26.3 ± 1.2	27.6 ± 1.7	27 ± 1	0.5662			
**Infant Characteristics**							
Age (mo.)	5.0 ± 0.4	4.4 ± 0.1	4.7 ± 0.2	0.1516	5.5 ± 1.5	*640*	From: https://doi.org/10.1016/j.jpeds.2017.01.014
Weight (kg)	6.9 ± 0.2	6.7 ± 0.2	6.8 ± 0.1	0.5732	6.9 ± 1.0	*640*	From: https://doi.org/10.1016/j.jpeds.2017.01.014
BMI (kg/m^2)	16.9 ± 0.5	16.6 ± 0.3	16.7 ± 0.3	0.5646			
Fat mass (kg)	1.7 ± 0.1	1.7 ± 0.1	1.7 ± 0.1	0.9646	1.65 ± 0.49	*640*	From: https://doi.org/10.1016/j.jpeds.2017.01.014
Fat mass (%)	24.4 ± 1.7	25.3 ± 1.2	24.9 ± 1	0.6771	24.0 ± 5.4	*640*	From: https://doi.org/10.1016/j.jpeds.2017.01.014
Fat free mass (kg)	5.2 ± 0.2	5 ± 0.1	5.1 ± 0.1	0.4571	5.15 ± 0.65	*640*	From: https://doi.org/10.1016/j.jpeds.2017.01.014
Fat free mass (%)	75.6 ± 1.7	74.7 ± 1.2	75.1 ± 1	0.6771			
**Child Characteristics**							

Age (yrs)	4.9 ± 0.2	4.5 ± 0.1	4.7 ± 0.1	0.0909	4.8 ± 0.7	*419*	From: https://doi.org/10.1210/clinem/dgab115
Weight (kg)	17.5 ± 0.3	16.8 ± 0.7	17.1 ± 0.4	0.3712	18.3 ± 3.2	*419*	From: https://doi.org/10.1210/clinem/dgab115
BMI (kg/m^2)	15.3 ± 0.5	15.3 ± 0.2	15.3 ± 0.2	0.9563	15.5 ± 1.6	*419*	From: https://doi.org/10.1210/clinem/dgab115
Fat mass (kg)	3.2 ± 0.5	3.4 ± 0.5	3.3 ± 0.3	0.8530	3.7 ± 1.7	*419*	From: https://doi.org/10.1210/clinem/dgab115
Fat mass (%)	18.3 ± 2.7	20.3 ± 2.7	19.4 ± 1.8	0.6019	19.9 ± 6.6	*419*	From: https://doi.org/10.1210/clinem/dgab115
Fat free mass (kg)	14.2 ± 0.2	13.3 ± 0.7	13.7 ± 0.4	0.2045	14.6 ± 2.5	*419*	From: https://doi.org/10.1210/clinem/dgab115
Fat free mass (%)	81.7 ± 2.7	79.7 ± 2.7	80.6 ± 1.8	0.6031			
Waist circumference (cm)	51.2 ± 1.1	50.6 ± 0.5	50.9 ± 0.6	0.5881			

Data are mean ± SEM, unless otherwise stated. *P<0.05 in T-Test or Chi-Square or Fisher's Exact test where appropriate

Please note full Healthy Start Cohort data from published reports are mean ± SD, unless otherwise noted;
#reported as median (25th–75th percentiles)

Table S2. Antibodies for simple western

Target	Molecular Weight (kDa)	Antibody	Antibody Dilution	Protein concentration (mg/mL)
AMPK	61	Cell Signaling (#2532)	1:50	0.2
AMPK (Thr172)	61	Cell Signaling (#2535)	1:50	0.2
ACC	220	Cell Signaling (#3662)	0.180555556	0.2
ACC (Ser 79)	220	Cell Signaling (#3661)	0.180555556	0.2

Table S3. Markers of myogenic differentiation and characteristics by Cluster

Cluster	MHC Mean	95% CL		P-value
1	1.03	0.79	1.28	0.6332
2	0.88	0.64	1.13	
3	1.00	0.81	1.19	

Cluster	Myogenin Mean	95% CL		P-value
1	0.98	0.78	1.17	0.6154
2	0.84	0.62	1.06	
3	0.93	0.76	1.11	

Cluster	Pax7 Mean	95% CL		P-value
1	0.91	0.72	1.1	0.4043
2	0.76	0.56	0.96	
3	0.91	0.75	1.07	

Cluster	%Cells undergoing myogenesis Mean	95% CL		P-value
1	79.54	69.91	89.17	0.2937
2	76.39	64.21	88.57	
3	69.52	60.45	78.6	

Cluster	Passage # Mean	95% CL		P-value
1	4.88	4.14	5.61	0.3379
2	4.88	4.14	5.61	
3	4.31	3.73	4.88	

Table S4. Features with ANOVA P<0.05 between lipid challenge conditions, all infants

Feature	f.value	p.value	-LOG10(p)	FDR	Fisher's LSD
CAR(16:0)	110.93	5.16E-25	24.287	6.40E-23	2 - 1; 3 - 1; 2 - 3
CAR(12:0)	95.126	6.33E-23	22.199	2.66E-21	2 - 1; 3 - 1; 2 - 3
CAR(16:1)	95.078	6.43E-23	22.192	2.66E-21	2 - 1; 3 - 1; 2 - 3
CAR(18:1)	92.283	1.59E-22	21.798	4.93E-21	2 - 1; 3 - 1; 2 - 3
CAR(14:0)	56.903	1.06E-16	15.974	2.63E-15	2 - 1; 3 - 1; 2 - 3
TG(56:4)	42.162	1.20E-13	12.92	2.48E-12	2 - 1; 3 - 1
CAR(18:0)	41.851	1.41E-13	12.85	2.50E-12	2 - 1; 3 - 1
TG(54:3)	41.099	2.09E-13	12.68	3.24E-12	2 - 1; 3 - 1; 2 - 3
TG(52:2)	39.452	4.98E-13	12.303	6.86E-12	2 - 1; 3 - 1; 2 - 3
TG(50:1)	34.585	7.20E-12	11.143	8.92E-11	2 - 1; 3 - 1; 2 - 3
TG(54:2)	33.791	1.13E-11	10.947	1.27E-10	2 - 1; 3 - 1; 2 - 3
TG(56:5)	28.535	2.53E-10	9.5976	2.61E-09	2 - 1; 3 - 1; 2 - 3
TG(56:3)	27.982	3.55E-10	9.4501	3.38E-09	2 - 1; 3 - 1
TG(48:0)	23.016	8.45E-09	8.073	7.49E-08	2 - 1; 3 - 1; 2 - 3
TG(52:1)	21.854	1.84E-08	7.7361	1.51E-07	2 - 1; 3 - 1; 2 - 3
TG(52:3)	21.766	1.95E-08	7.7105	1.51E-07	2 - 1; 3 - 1; 2 - 3
DG(18:1/18:1/0:0)	20.66	4.13E-08	7.384	3.01E-07	2 - 1; 3 - 1; 2 - 3
TG(50:2)	19.701	8.01E-08	7.0965	5.52E-07	2 - 1; 3 - 1; 2 - 3
DG(16:0/20:1/0:0)	18.027	2.61E-07	6.5841	1.70E-06	2 - 1; 3 - 1; 2 - 3
DG(16:0/18:1/0:0)	15.577	1.55E-06	5.8091	9.62E-06	2 - 1; 3 - 1; 2 - 3
DG(16:0/16:0/0:0)	15.31	1.89E-06	5.7231	1.12E-05	2 - 1; 3 - 1; 2 - 3
TG(54:4)	14.526	3.41E-06	5.4674	1.92E-05	2 - 1; 3 - 1
TG(56:6)	12.889	1.20E-05	4.9222	6.45E-05	2 - 1; 3 - 1
TG(48:1)	12.578	1.52E-05	4.8168	7.88E-05	2 - 1; 2 - 3
SM(24:3)	10.608	7.30E-05	4.1365	0.00035919	1 - 2; 1 - 3
TG(54:1)	10.534	7.75E-05	4.1105	0.00035919	2 - 1; 3 - 1
SM(14:0)	10.523	7.82E-05	4.1067	0.00035919	1 - 2; 1 - 3
TG(56:7)	10.309	9.30E-05	4.0313	0.00041204	2 - 1; 3 - 1
DG(16:0/20:2/0:0)	9.2571	0.00022081	3.656	0.00091958	2 - 1; 2 - 3
TG(50:0)	9.248	0.00022248	3.6527	0.00091958	2 - 1; 3 - 1
DG(18:1/0:0/18:1)	8.2535	0.00051158	3.2911	0.0020463	2 - 1; 3 - 1
TG(54:5)	6.9266	0.0015923	2.798	0.0061702	2 - 1; 3 - 1
DG(16:0/20:3/0:0)	6.8761	0.0016635	2.779	0.0062507	2 - 1; 3 - 1
SM(24:2)	6.6166	0.0020848	2.6809	0.0076033	1 - 2; 1 - 3
DG(16:0/20:0/0:0)	5.8663	0.0040292	2.3948	0.014275	2 - 1; 3 - 1
TG(52:4)	5.1993	0.0072971	2.1368	0.025134	2 - 1; 3 - 1
DG(18:0/18:1/0:0)	4.9781	0.0089015	2.0505	0.029832	2 - 1; 3 - 1
DG(14:0/16:1/0:0)	4.9163	0.0094118	2.0263	0.030712	1 - 2; 1 - 3
SM(20:1)	4.3886	0.015182	1.8187	0.047136	1 - 3
TG(50:3)	4.387	0.015205	1.818	0.047136	2 - 1

1 = BSA
2 = 24hFA
3 = FA+RF

Table S5. Features with ANOVA P<0.05 between lipid challenge conditions, Cluster 1

Feature	f.value	p.value	-LOG10(p)	FDR	Fisher's LSD
CAR(16:0)	29.545	3.37E-07	6.4721	2.60E-05	24hFA - BSA; 24hFA - FARF; FARF - BSA
CAR(12:0)	28.801	4.19E-07	6.3779	2.60E-05	24hFA - BSA; 24hFA - FARF; FARF - BSA
TG(56:4)	23.844	1.98E-06	5.7029	8.19E-05	24hFA - BSA; FARF - BSA
CAR(18:1)	20.479	6.47E-06	5.1891	0.00019	24hFA - BSA; 24hFA - FARF; FARF - BSA
CAR(16:1)	20.021	7.67E-06	5.1151	0.00019	24hFA - BSA; FARF - BSA
TG(56:3)	19.242	1.03E-05	4.9866	0.000195	24hFA - BSA; FARF - BSA
DG(16:0/20:1/0:0)	19.071	1.10E-05	4.9581	0.000195	24hFA - BSA; 24hFA - FARF; FARF - BSA
TG(54:3)	16.693	2.86E-05	4.5432	0.00043	24hFA - BSA; 24hFA - FARF; FARF - BSA
TG(52:2)	16.487	3.12E-05	4.5056	0.00043	24hFA - BSA; 24hFA - FARF; FARF - BSA
TG(54:2)	15.616	4.53E-05	4.3438	0.000522	24hFA - BSA; 24hFA - FARF; FARF - BSA
TG(50:1)	15.567	4.63E-05	4.3345	0.000522	24hFA - BSA; 24hFA - FARF; FARF - BSA
TG(56:5)	14.261	8.29E-05	4.0815	0.000856	24hFA - BSA; FARF - BSA
TG(52:1)	12.025	0.00024106	3.6179	0.002299	24hFA - BSA; 24hFA - FARF; FARF - BSA
CAR(18:0)	10.132	0.00064569	3.19	0.005719	24hFA - BSA; FARF - BSA
TG(48:0)	9.159	0.0011072	2.9558	0.009153	24hFA - BSA; 24hFA - FARF
CAR(14:0)	8.6061	0.0015214	2.8178	0.011791	24hFA - BSA; FARF - BSA
TG(52:3)	7.8399	0.0023972	2.6203	0.017486	24hFA - BSA
TG(50:2)	7.5401	0.0028778	2.5409	0.019825	24hFA - BSA; 24hFA - FARF
DG(18:1/18:1/0:0)	7.2373	0.0034709	2.4596	0.022652	24hFA - BSA
DG(16:1/0:0/18:1)	6.446	0.0057458	2.2407	0.035624	24hFA - FARF; BSA - FARF
TG(54:4)	6.2814	0.0063983	2.1939	0.037781	24hFA - BSA
TG(50:0)	6.1827	0.0068279	2.1657	0.038484	24hFA - BSA; 24hFA - FARF

Table S6. Features with ANOVA P<0.05 between lipid challenge conditions, Cluster 2

Feature	f.value	p.value	-LOG10(p)	FDR	Fisher's LSD
CAR(12:0)	32.516	1.47E-07	6.832	1.02E-05	24hFA - BSA; 24hFA - FARF; FARF - BSA
CAR(16:0)	32.095	1.65E-07	6.7825	1.02E-05	24hFA - BSA; 24hFA - FARF; FARF - BSA
CAR(18:1)	28.978	3.98E-07	6.4005	1.64E-05	24hFA - BSA; 24hFA - FARF; FARF - BSA
CAR(16:1)	23.262	2.41E-06	5.6175	7.48E-05	24hFA - BSA; 24hFA - FARF; FARF - BSA
CAR(14:0)	20.685	6.00E-06	5.222	0.00014875	24hFA - BSA; 24hFA - FARF; FARF - BSA
TG(50:1)	11.584	0.00030112	3.5213	0.0062231	24hFA - BSA; 24hFA - FARF; FARF - BSA
CAR(18:0)	11.124	0.00038137	3.4186	0.0067558	24hFA - BSA; FARF - BSA
TG(48:0)	9.9797	0.00070133	3.1541	0.010871	24hFA - BSA; FARF - BSA
TG(52:2)	9.5641	0.00088184	3.0546	0.01215	24hFA - BSA; FARF - BSA
DG(16:0/16:0/0:0)	8.1917	0.0019413	2.7119	0.024073	24hFA - BSA; 24hFA - FARF
TG(54:3)	7.9	0.0023118	2.6361	0.02606	24hFA - BSA; FARF - BSA
TG(52:1)	7.5803	0.0028076	2.5517	0.029012	24hFA - BSA; FARF - BSA
TG(54:2)	7.3549	0.0032262	2.4913	0.030773	24hFA - BSA; FARF - BSA
TG(56:4)	7.092	0.0038016	2.42	0.033671	24hFA - BSA; FARF - BSA

Table S7. Features with ANOVA P<0.05 between lipid challenge conditions, Cluster 3

Feature	f.value	p.value	-LOG10(p)	FDR	Fisher's LSD
CAR(16:0)	70.457	3.58E-13	12.446	4.44E-11	24hFA - BSA; 24hFA - FARF; FARF - BSA
CAR(16:1)	56.227	8.41E-12	11.075	5.21E-10	24hFA - BSA; 24hFA - FARF; FARF - BSA
CAR(18:1)	48.806	5.60E-11	10.252	2.32E-09	24hFA - BSA; 24hFA - FARF; FARF - BSA
CAR(14:0)	33.674	5.70E-09	8.2441	1.46E-07	24hFA - BSA; 24hFA - FARF; FARF - BSA
CAR(12:0)	33.574	5.90E-09	8.2289	1.46E-07	24hFA - BSA; 24hFA - FARF; FARF - BSA
CAR(18:0)	27.546	5.53E-08	7.2572	1.14E-06	24hFA - BSA; FARF - BSA
TG(54:3)	19.612	1.73E-06	5.7611	3.07E-05	24hFA - BSA; 24hFA - FARF; FARF - BSA
TG(56:4)	17.654	4.54E-06	5.343	6.53E-05	24hFA - BSA; FARF - BSA
TG(52:2)	17.569	4.74E-06	5.3243	6.53E-05	24hFA - BSA; 24hFA - FARF; FARF - BSA
TG(54:2)	13.8	3.56E-05	4.4487	0.000402	24hFA - BSA; FARF - BSA
TG(50:1)	13.796	3.57E-05	4.4479	0.000402	24hFA - BSA; 24hFA - FARF; FARF - BSA
TG(52:3)	12.967	5.74E-05	4.2412	0.000593	24hFA - BSA; 24hFA - FARF; FARF - BSA
TG(56:5)	11.395	0.000147	3.834	0.001398	24hFA - BSA; FARF - BSA
TG(54:4)	9.7303	0.000419	3.3783	0.003707	24hFA - BSA; FARF - BSA
TG(50:2)	9.4971	0.000487	3.3123	0.004028	24hFA - BSA; 24hFA - FARF
DG(16:0/16:0/0:0)	9.1387	0.000617	3.2097	0.004773	24hFA - BSA; FARF - BSA
TG(56:3)	9.0502	0.000654	3.1842	0.004773	24hFA - BSA; FARF - BSA
TG(48:0)	8.4932	0.000952	3.0215	0.006556	24hFA - BSA; 24hFA - FARF
TG(56:6)	8.2696	0.001109	2.9553	0.007235	24hFA - BSA; FARF - BSA
TG(52:1)	7.9703	0.001362	2.8657	0.008447	24hFA - BSA; FARF - BSA
DG(18:1/18:1/0:0)	7.2495	0.002261	2.6457	0.013352	24hFA - BSA; FARF - BSA
DG(16:0/18:1/0:0)	6.9771	0.002749	2.5608	0.015494	24hFA - BSA; FARF - BSA
DG(16:0/0:0/18:1)	6.0773	0.005321	2.274	0.028685	24hFA - BSA
DG(16:0/20:1/0:0)	5.8087	0.006511	2.1863	0.033641	24hFA - BSA; FARF - BSA
TG(48:1)	5.4275	0.008707	2.0601	0.043185	24hFA - BSA; 24hFA - FARF
TG(54:5)	5.3461	0.00927	2.0329	0.044209	24hFA - BSA
TG(56:7)	5.2723	0.009813	2.0082	0.045065	24hFA - BSA; FARF - BSA

Table S8. Individual lipid species across lipid challenge conditions, with unadjusted t-test between normal weight or obese pregnancies conditions, all infants

Myo-MSC condition			
**Feature**	**NW, mean ± SEM**	**Ob, mean ± SEM**	**p.value**
CAR(12:0)	0.23 ± 0.04	0.43 ± 0.13	0.14577701
CAR(14:0)	1.33 ± 0.24	1.61 ± 0.52	0.62642335
CAR(16:0)	4.36 ± 0.59	6.69 ± 1.75	0.22798262
CAR(16:1)	1.12 ± 0.31	1.26 ± 0.34	0.76612166
CAR(18:0)	3.88 ± 0.83	7.15 ± 2.67	0.26559139
CAR(18:1)	4.66 ± 0.99	7.73 ± 2.92	0.33977848
Sum CAR	15.58 ± 2.73	24.88 ± 7.56	0.2689681
SM(14:0)	797.48 ± 56.54	780.28 ± 49.51	0.82006621
SM(16:1)	271.77 ± 38.1	296.79 ± 33.2	0.62310658
SM(18:1)	87.52 ± 10.73	87.72 ± 5.62	0.98671277
SM(18:0)	734.4 ± 84.42	665.9 ± 58.92	0.50668344
SM(22:0)	1150.28 ± 91.1	1031.16 ± 116.36	0.43092239
SM(20:0)	276.31 ± 29.37	252.02 ± 28.29	0.55596876
SM(23:0)	389.44 ± 34.2	377.08 ± 53.49	0.84919992
SM(24:0)	1791.21 ± 146.7	1650.58 ± 183.28	0.55707105
SM(24:2)	1088.63 ± 125.51	1091.03 ± 102.74	0.9882088
SM(24:3)	123.72 ± 10.95	120.36 ± 9.01	0.81329549
SM(24:4)	20.09 ± 1.76	19.82 ± 1.44	0.90746888
SM(18:2)	3.47 ± 0.31	2.63 ± 0.26	0.04296595
SM(20:1)	66.31 ± 5.17	57.87 ± 6.58	0.32569683
SM(20:2)	6.32 ± 0.47	5.5 ± 0.48	0.2378192
SM(20:3)	3.37 ± 0.5	2.97 ± 0.37	0.52154361
SM(22:1)	877.12 ± 55.82	719.48 ± 101.07	0.19057719
Sum SM	7687.42 ± 580.93	7161.2 ± 608.06	0.53743121
GlcCer(20:0)	107.27 ± 33.75	163.6 ± 42.09	0.30896357
GlcCer(23:0)	81.6 ± 22	131.36 ± 38.49	0.27916434
GlcCer(22:0)	122.79 ± 48.36	157.84 ± 50.75	0.62180848
GlcCer(16:0)	90.13 ± 28.99	145.34 ± 31.97	0.21289029
GlcCer(24:0)	154.58 ± 54.47	84.37 ± 27.43	0.25018195
GlcCer(18:0)	105.44 ± 25.39	107.15 ± 30.85	0.96645932
GlcCer(24:1)	127.84 ± 30.72	76.71 ± 27.86	0.22651055
Sum GluCer	789.66 ± 121.14	866.37 ± 106.21	0.63642236
LacCer(20:0)	12.89 ± 4.18	38.71 ± 21.01	0.25203326
LacCer(23:0)	59.38 ± 19.76	185.31 ± 90.47	0.19751581
LacCer(18:0)	30.12 ± 9.33	85.65 ± 41.42	0.21459048
SPB(18:1;O2)	90.73 ± 13.97	109.16 ± 17.98	0.42905103
SPB(18:1;O)	51.29 ± 6.67	40.8 ± 5.77	0.24261705
SPBP(18:1;O2)	2.05 ± 1.03	2.92 ± 1.11	0.57175379
Cer(14:0)	15.14 ± 1.66	13.23 ± 1.85	0.45054249
Cer(16:0)	345.38 ± 54.75	320.77 ± 48.17	0.73734173
Cer(18:0)	59.47 ± 9.33	46.81 ± 7.75	0.30306503
Cer(20:0)	15.94 ± 2.69	12.77 ± 2.2	0.36682228
Cer(22:0)	71.91 ± 11.57	65.67 ± 12.44	0.71707949
Cer(23:0)	24.81 ± 3.64	24.8 ± 4.89	0.99921802
Cer(24:0)	133.72 ± 16.88	135.87 ± 23.9	0.94280163
Cer(24:1)	260.28 ± 32.93	277.28 ± 36.02	0.73122396
Cer(24:2)	17.54 ± 2.16	17.75 ± 2.34	0.9479211
Cer(26:0)	1.37 ± 0.18	1.26 ± 0.18	0.67470595
Cer(26:1)	4.01 ± 0.44	3.96 ± 0.44	0.94605238
Sum Cer	949.57 ± 126.76	920.18 ± 134.52	0.8751445
Cer(16:0;O2)	66.72 ± 18.33	43.66 ± 10.45	0.27574815
Cer(18:0;O2)	15.56 ± 4.1	10.3 ± 3.86	0.3569041
Cer(20:0;O2)	12.07 ± 3.21	7.07 ± 2.53	0.22755851
Cer(22:0;O2)	26.88 ± 7.61	13.34 ± 4.67	0.1351962
Cer(23:0;O2)	5.66 ± 1.59	3.17 ± 1	0.18848501
Cer(24:0;O2)	20.85 ± 5.68	10.46 ± 3.12	0.11358379
Cer(26:0;O2)	0.42 ± 0.13	0.22 ± 0.05	0.1650324
Cer(26:1;O2)	1.61 ± 0.36	0.84 ± 0.34	0.13678144
Sum O2Cer	149.78 ± 37.18	89.06 ± 25.28	0.18249151
TG(48:2)	339.25 ± 68.13	447.73 ± 88.53	0.34404334
TG(48:1)	585.68 ± 111.76	716.59 ± 129.35	0.4526229
TG(48:0)	208.67 ± 39.56	252.05 ± 49.91	0.50482679
TG(50:3)	406.78 ± 101.35	525.61 ± 111.94	0.43970662
TG(50:2)	1350.09 ± 304.17	1628.61 ± 311.06	0.52781843
TG(50:1)	1187.79 ± 279.72	1301.44 ± 240.7	0.7593836

TG(50:0)	165.23 ± 40.4	197.03 ± 39.99	0.58051769
TG(52:5)	153.74 ± 44.55	266.85 ± 69.45	0.18721186
TG(52:4)	384.92 ± 99.76	541.03 ± 126.12	0.34381976
TG(52:3)	1435.49 ± 358.04	1716.03 ± 305.31	0.55399815
TG(52:2)	2905.43 ± 742	3052.17 ± 478.15	0.86744649
TG(52:1)	709.6 ± 200.8	823.18 ± 147.54	0.64898289
TG(52:0)	61.74 ± 14.55	68.82 ± 13.22	0.72065264
TG(54:6)	294.5 ± 80.96	449.16 ± 88.18	0.20829944
TG(54:5)	553.28 ± 151.57	736.9 ± 149.17	0.39522828
TG(54:4)	855.38 ± 203.8	1035.97 ± 165.97	0.49504126
TG(54:3)	2147.82 ± 494.63	2299.43 ± 346.47	0.80152289
TG(54:2)	1219.95 ± 281.34	1428.75 ± 209.97	0.55324895
TG(54:1)	48.27 ± 15.34	62.42 ± 13.48	0.49264315
TG(56:7)	668.63 ± 189.8	961.79 ± 162.39	0.24819253
TG(56:6)	655.58 ± 165.27	882.32 ± 128.23	0.28388511
TG(56:5)	554.97 ± 117.76	709.84 ± 110.55	0.34505516
TG(56:4)	540.89 ± 126.36	574.55 ± 90.1	0.82814639
TG(56:3)	130.55 ± 34.59	145.26 ± 24.17	0.72714738
Sum TG	17564.2 ± 4158.97	20823.52 ± 3346.91	0.54382259
DG(14:0/14:0/0:0)	1.95 ± 0.45	1.4 ± 0.36	0.33983202
DG(14:0/16:1/0:0)	5.76 ± 0.99	4.98 ± 0.97	0.58131279
DG(14:0/16:0/0:0)	23.99 ± 3.09	18.19 ± 3.03	0.19045445
DG(16:1/16:1/0:0)	6.11 ± 1.11	5.2 ± 0.73	0.49610512
DG(14:0/18:1/0:0)	21.73 ± 3.3	18.95 ± 2.93	0.53164292
DG(16:0/16:1/0:0)	49.9 ± 7.81	38.87 ± 4.41	0.22119892
DG(14:0/18:0/0:0)	2.51 ± 0.52	2.01 ± 0.32	0.41855227
DG(16:0/16:0/0:0)	47.38 ± 6.82	36.66 ± 3.53	0.16549137
DG(16:0/18:2/0:0)	17.35 ± 3.39	14.28 ± 1.77	0.41954748
DG(16:1/18:1/0:0)	74.89 ± 11.45	67.55 ± 8.21	0.6026905
DG(16:0/18:1/0:0)	216.78 ± 38.92	184.74 ± 19.59	0.45957325
DG(18:0/16:1/0:0)	22.31 ± 3.31	21.18 ± 1.95	0.7663366
DG(16:0/18:0/0:0)	26.68 ± 5.95	23.61 ± 2.53	0.63090746
DG(16:0/20:4/0:0)	23.55 ± 3.11	22.86 ± 2.1	0.85409567
DG(18:2/18:2/0:0)	1.11 ± 0.31	1.05 ± 0.18	0.86455487
DG(16:0/20:3/0:0)	8.46 ± 1.48	7.73 ± 0.84	0.66692127
DG(18:0/18:3/0:0)	1.74 ± 0.41	1.45 ± 0.18	0.51124163
DG(18:2/18:1/0:0)	30.84 ± 5.03	27.39 ± 3.47	0.57283438
DG(16:0/20:2/0:0)	7.06 ± 1.64	6.35 ± 0.63	0.68421361
DG(18:0/18:2/0:0)	25.16 ± 2.94	21.68 ± 2.34	0.35948325
DG(18:1/18:1/0:0)	169.71 ± 27.18	152.12 ± 18.07	0.58969704
DG(16:0/20:1/0:0)	14.51 ± 3.99	10.45 ± 1.28	0.32650795
DG(18:0/18:1/0:0)	146.11 ± 18.62	136.4 ± 13.18	0.67037672
DG(16:0/20:0/0:0)	1 ± 0.27	0.93 ± 0.2	0.84987837
DG(18:0/18:0/0:0)	9.14 ± 1.38	7.6 ± 0.79	0.33152879
DG(16:0/22:6/0:0)	16.61 ± 3.93	18.49 ± 2.27	0.67682189
DG(18:0/20:4/0:0)	272.14 ± 33.05	226 ± 36.6	0.35932117
DG(18:0/22:6/0:0)	17.8 ± 1.76	15.49 ± 1.67	0.34885898
DG(14:0/0:0/16:0)	6.81 ± 1.73	10.8 ± 5.1	0.47643676
DG(14:0/0:0/18:1)	5.91 ± 1.67	8.62 ± 3.5	0.49901082
DG(16:0/0:0/16:1)	12.43 ± 2.67	20.36 ± 5.97	0.24552029
DG(16:0/0:0/16:0)	19.24 ± 3.38	23.02 ± 3.91	0.47306932
DG(16:0/0:0/18:2)	6.2 ± 1.44	11.12 ± 2.25	0.08030475
DG(16:1/0:0/18:1)	22.08 ± 4.09	26.99 ± 8.64	0.61881555
DG(16:0/0:0/18:1)	55.47 ± 9.62	80.7 ± 25.94	0.3812367
DG(18:0/0:0/16:1)	9.57 ± 3.33	7.85 ± 1.81	0.64842137
DG(16:0/0:0/18:0)	32.52 ± 6.32	45.76 ± 9.04	0.24509676
DG(16:0/0:0/20:4)	10.22 ± 2.58	13.65 ± 4.61	0.52839431
DG(18:2/0:0/18:1)	7.32 ± 2.2	10.42 ± 2.5	0.36220522
DG(18:0/0:0/18:2)	8.13 ± 1.83	8.48 ± 1.64	0.88764391
DG(18:1/0:0/18:1)	49.41 ± 13.37	64.76 ± 21.39	0.55354935
DG(18:0/0:0/18:1)	50.85 ± 7.92	68.61 ± 24.1	0.50080016
DG(18:0/0:0/18:0)	39.31 ± 10.01	50.74 ± 15.31	0.5427575
DG(16:0/0:0/22:6)	6.93 ± 2.55	18.41 ± 6.46	0.11765416
DG(18:0/0:0/20:4)	157.04 ± 23.97	213.87 ± 63.98	0.42414456
DG(18:0/0:0/22:6)	9.18 ± 1.53	18.87 ± 5.19	0.0926333
Sum DG	1770.88 ± 218.43	1796.66 ± 247.63	0.93863778

BSA condition

Feature	NW, mean ± SEM	Ob, mean ± SEM	p.value
CAR(12:0)	0.22 ± 0.04	0.15 ± 0.03	0.22587529

CAR(14:0)	0.99 ± 0.28	0.58 ± 0.11	0.178065697
CAR(16:0)	3.73 ± 0.81	2.91 ± 0.5	0.389538471
CAR(16:1)	0.84 ± 0.23	0.53 ± 0.09	0.215129139
CAR(18:0)	2.86 ± 0.73	2.36 ± 0.52	0.576995967
CAR(18:1)	3.52 ± 0.88	1.86 ± 0.28	0.07593083
Sum CAR	12.15 ± 2.85	8.4 ± 1.37	0.235987517
SM(14:0)	793.79 ± 48.75	767.87 ± 34.75	0.665180035
SM(16:1)	277.81 ± 25.33	283.78 ± 29.22	0.879057601
SM(18:1)	92.15 ± 11.96	83.91 ± 6.63	0.544903983
SM(18:0)	710.42 ± 85.27	645.53 ± 62.91	0.541517183
SM(22:0)	1070.62 ± 86.22	997.29 ± 99.74	0.584566652
SM(20:0)	254.91 ± 20.73	239.27 ± 26.71	0.650190627
SM(23:0)	353 ± 25.68	352.49 ± 38.9	0.991495563
SM(24:0)	1647.33 ± 166.41	1568.2 ± 156.56	0.731358853
SM(24:2)	1074.57 ± 124.71	1035.41 ± 96.33	0.804076995
SM(24:3)	123.04 ± 8.87	120.42 ± 9.43	0.841771053
SM(24:4)	19.51 ± 1.08	19.41 ± 1.17	0.949347358
SM(18:2)	2.96 ± 0.29	2.55 ± 0.23	0.279925267
SM(20:1)	66.21 ± 4.77	57.46 ± 5.63	0.248979081
SM(20:2)	6.4 ± 0.31	5.78 ± 0.42	0.252158981
SM(20:3)	3.22 ± 0.29	2.89 ± 0.26	0.410831451
SM(22:1)	864.42 ± 75.95	722.29 ± 62.27	0.156387804
Sum SM	7360.33 ± 571.48	6904.57 ± 542.41	0.567175602
GlcCer(20:0)	99.36 ± 27.26	123.53 ± 40.73	0.630274934
GlcCer(23:0)	88.74 ± 31.18	142.56 ± 41.59	0.314000142
GlcCer(22:0)	122.62 ± 39.55	127.95 ± 38.74	0.923938925
GlcCer(16:0)	73.42 ± 17.93	119.86 ± 29.17	0.192358654
GlcCer(24:0)	158.94 ± 50.38	112.63 ± 31.17	0.434481185
GlcCer(18:0)	89.1 ± 15.85	74.58 ± 19.71	0.573585465
GlcCer(24:1)	133.67 ± 36.18	113.73 ± 33.96	0.690445666
Sum GluCer	765.86 ± 94.62	814.86 ± 105.88	0.733742343
LacCer(20:0)	23.28 ± 9.38	19.55 ± 6.76	0.746854705
LacCer(23:0)	115.39 ± 53.85	95.83 ± 33.36	0.756406214
LacCer(18:0)	55.21 ± 21.99	42.38 ± 13.38	0.616821958
SPB(18:1;O2)	64.39 ± 8.21	59.22 ± 7.92	0.654170097
SPB(18:1;O)	34.78 ± 8.67	27.98 ± 4.05	0.47432572
SPBP(18:1;O2)	1.53 ± 0.4	1.69 ± 0.47	0.796094223
Cer(14:0)	17.48 ± 1.48	16.18 ± 1.65	0.561993624
Cer(16:0)	370.86 ± 43.06	362.54 ± 42.02	0.891036145
Cer(18:0)	65.49 ± 8.01	59.91 ± 10.61	0.680656602
Cer(20:0)	16.92 ± 2.21	16.4 ± 3.02	0.891371225
Cer(22:0)	72.54 ± 10.6	73.98 ± 11.63	0.928418129
Cer(23:0)	24.41 ± 3.31	26.62 ± 4.07	0.679045112
Cer(24:0)	134.77 ± 15.02	148.17 ± 21.49	0.617429131
Cer(24:1)	257.28 ± 27.94	281.19 ± 35.62	0.604597685
Cer(24:2)	18.48 ± 2.04	19.82 ± 2.5	0.683494982
Cer(26:0)	1.51 ± 0.17	1.43 ± 0.22	0.790873101
Cer(26:1)	4.26 ± 0.37	4.6 ± 0.58	0.631400753
Sum Cer	984.01 ± 108.16	1010.84 ± 126.34	0.873751698
Cer(16:0;O2)	65.81 ± 13.66	47.74 ± 8.7	0.26756011
Cer(18:0;O2)	15.04 ± 3.04	10.65 ± 2.71	0.287998959
Cer(20:0;O2)	12.75 ± 2.92	7.82 ± 1.97	0.166737006
Cer(22:0;O2)	26.63 ± 6.12	15.18 ± 3.63	0.113368724
Cer(23:0;O2)	5.92 ± 1.25	3.32 ± 0.75	0.080394557
Cer(24:0;O2)	19.87 ± 4.02	11.91 ± 2.52	0.09937772
Cer(26:0;O2)	0.37 ± 0.07	0.27 ± 0.07	0.325430978
Cer(26:1;O2)	1.16 ± 0.2	0.91 ± 0.41	0.607495164
Sum O2Cer	147.55 ± 28.4	97.8 ± 19.75	0.156798978
TG(48:2)	362.37 ± 33.95	531.21 ± 91.15	0.101398067
TG(48:1)	625.75 ± 56.12	857.86 ± 122.87	0.103663534
TG(48:0)	211.23 ± 13.86	267.65 ± 34.84	0.152855652
TG(50:3)	406.59 ± 44.15	547.46 ± 91.49	0.185124719
TG(50:2)	1311.39 ± 146.11	1766.32 ± 272.76	0.15994387
TG(50:1)	1106.55 ± 128.08	1332.25 ± 186.95	0.333688681

TG(50:0)	157.23 ± 22.52	207.63 ± 31.9	0.212430658
TG(52:5)	143.98 ± 22.3	250.15 ± 48.22	0.060358046
TG(52:4)	368.35 ± 46.37	521.07 ± 94.36	0.165591456
TG(52:3)	1308.65 ± 170.5	1688.12 ± 278.55	0.262201795
TG(52:2)	2457.49 ± 355.28	3035.9 ± 451.7	0.326726956
TG(52:1)	620.31 ± 103.64	751.73 ± 109.4	0.391674376
TG(52:0)	56.56 ± 10.59	72.37 ± 13.84	0.376433717
TG(54:6)	267.4 ± 47.55	419.95 ± 87.18	0.14246294
TG(54:5)	493.04 ± 82.44	766.49 ± 150.65	0.128953627
TG(54:4)	758.47 ± 107.07	1011.59 ± 155.69	0.196351356
TG(54:3)	1791.34 ± 251.98	2187.67 ± 301.57	0.324867611
TG(54:2)	1005.21 ± 136.54	1221.24 ± 149.09	0.295963687
TG(54:1)	49.64 ± 11.83	53.51 ± 9.93	0.802979004
TG(56:7)	626.81 ± 117.14	894.25 ± 153.91	0.181477413
TG(56:6)	596.69 ± 84.81	831.42 ± 123.68	0.13316573
TG(56:5)	501.6 ± 59.35	627.74 ± 72.26	0.191028136
TG(56:4)	440.93 ± 59.16	475.5 ± 50.28	0.658011808
TG(56:3)	117.26 ± 22.81	126.52 ± 16.05	0.739819002
Sum TG	15784.83 ± 2008.66	20445.58 ± 2949.13	0.20753687
DG(14:0/14:0/0:0)	2.03 ± 0.44	1.65 ± 0.25	0.445171291
DG(14:0/16:1/0:0)	6.83 ± 1.04	5.72 ± 0.52	0.338990984
DG(14:0/16:0/0:0)	27.51 ± 3.08	23.23 ± 2.1	0.254556541
DG(16:1/16:1/0:0)	6.08 ± 0.73	5.54 ± 0.57	0.563848234
DG(14:0/18:1/0:0)	23.24 ± 2.39	21.82 ± 1.58	0.618250997
DG(16:0/16:1/0:0)	48.58 ± 4.07	45.51 ± 3.94	0.591363845
DG(14:0/18:0/0:0)	2.53 ± 0.26	2.19 ± 0.2	0.311740919
DG(16:0/16:0/0:0)	49.17 ± 3.83	44.92 ± 3.27	0.403790319
DG(16:0/18:2/0:0)	17.43 ± 1.56	16.96 ± 1.61	0.837527769
DG(16:1/18:1/0:0)	75.56 ± 7.3	70.18 ± 5.88	0.567796997
DG(16:0/18:1/0:0)	221.75 ± 24.93	207.94 ± 16.55	0.643937921
DG(18:0/16:1/0:0)	23.49 ± 2.58	20.73 ± 1.27	0.33577095
DG(16:0/18:0/0:0)	26.98 ± 4.4	25.57 ± 2.36	0.775776252
DG(16:0/20:4/0:0)	24.58 ± 2.67	23.48 ± 2.6	0.77034405
DG(18:2/18:2/0:0)	1.03 ± 0.19	0.82 ± 0.12	0.348459465
DG(16:0/20:3/0:0)	9.06 ± 1.43	8.09 ± 0.71	0.542076583
DG(18:0/18:3/0:0)	1.23 ± 0.11	1.5 ± 0.14	0.139681497
DG(18:2/18:1/0:0)	31.4 ± 3.43	28.96 ± 2.35	0.557749686
DG(16:0/20:2/0:0)	8.03 ± 1.33	6.82 ± 0.4	0.380806954
DG(18:0/18:2/0:0)	25.58 ± 1.82	22.88 ± 1.85	0.307464877
DG(18:1/18:1/0:0)	172.67 ± 20.26	151.98 ± 13.1	0.392408304
DG(16:0/20:1/0:0)	14.87 ± 2.69	12.62 ± 1.11	0.435480939
DG(18:0/18:1/0:0)	142.85 ± 14.82	130.68 ± 8.42	0.473997599
DG(16:0/20:0/0:0)	1.23 ± 0.25	0.97 ± 0.12	0.354533516
DG(18:0/18:0/0:0)	8.54 ± 0.96	8.26 ± 0.89	0.829281442
DG(16:0/22:6/0:0)	13.41 ± 1.95	12.95 ± 1.19	0.839342593
DG(18:0/20:4/0:0)	256.66 ± 29.14	190.39 ± 17.14	0.05601088
DG(18:0/22:6/0:0)	14.61 ± 1.54	11.63 ± 0.77	0.088305851
DG(14:0/0:0/16:0)	7.13 ± 1.41	12.51 ± 2.74	0.098300926
DG(14:0/0:0/18:1)	4.82 ± 1.38	5.53 ± 1.8	0.759367305
DG(16:0/0:0/16:1)	13.31 ± 2.29	17.5 ± 3.65	0.346425515
DG(16:0/0:0/16:0)	17.48 ± 2.65	23.46 ± 6.47	0.410753227
DG(16:0/0:0/18:2)	8.69 ± 1.51	7.95 ± 1.48	0.726339489
DG(16:1/0:0/18:1)	18.34 ± 3.13	26.62 ± 6.98	0.298936327
DG(16:0/0:0/18:1)	57.9 ± 7.95	67.49 ± 15.03	0.584335693
DG(18:0/0:0/16:1)	5.78 ± 1.22	8.1 ± 1.63	0.26601799
DG(16:0/0:0/18:0)	31.39 ± 6.89	51.16 ± 12.66	0.188674941
DG(16:0/0:0/20:4)	8.2 ± 1.61	8.68 ± 3.31	0.898512184
DG(18:2/0:0/18:1)	9.76 ± 2.57	8.16 ± 2.3	0.645310734
DG(18:0/0:0/18:2)	11.31 ± 2.77	8.45 ± 2.97	0.487743154
DG(18:1/0:0/18:1)	50.13 ± 12.15	57.07 ± 10.36	0.666077083
DG(18:0/0:0/18:1)	57.22 ± 10.82	36.33 ± 5.44	0.089329953
DG(18:0/0:0/18:0)	43.32 ± 9.97	58.15 ± 16.35	0.452152303
DG(16:0/0:0/22:6)	6.75 ± 1.34	10.39 ± 1.24	0.055585425
DG(18:0/0:0/20:4)	177.42 ± 38.46	139.23 ± 22.66	0.392181234

DG(18:0/0:0/22:6)	10.86 ± 3.22	12.5 ± 2.36	0.682069146
Sum DG	1796.72 ± 143.18	1663.25 ± 131.37	0.496849063

24hFA condition

Feature	NW, mean ± SEM	Ob, mean ± SEM	p.value
CAR(12:0)	3.37 ± 0.54	3.12 ± 0.51	0.737999364
CAR(14:0)	8.79 ± 1.49	7.07 ± 1.03	0.346670104
CAR(16:0)	29.26 ± 3.34	28.3 ± 3.87	0.852751162
CAR(16:1)	10.21 ± 1.2	8.88 ± 1.17	0.43461947
CAR(18:0)	13.57 ± 2.84	12.21 ± 2.06	0.697180596
CAR(18:1)	34.82 ± 4.93	31.5 ± 4.25	0.61233275
Sum CAR	100.01 ± 13.07	91.07 ± 11.4	0.60887322
SM(14:0)	606.24 ± 37.03	618.44 ± 37.5	0.818886983
SM(16:1)	299.79 ± 27.29	308.2 ± 24.84	0.820887703
SM(18:1)	91.15 ± 9.05	87.8 ± 7.75	0.779841287
SM(18:0)	729.18 ± 56.36	634.22 ± 60.15	0.260244988
SM(22:0)	1096.72 ± 69.94	955.91 ± 100.35	0.264887729
SM(20:0)	274.57 ± 18.83	240.62 ± 28.27	0.332281382
SM(23:0)	352.64 ± 23.7	325.2 ± 35.53	0.531191616
SM(24:0)	1625.26 ± 129.64	1415.25 ± 140.83	0.283412011
SM(24:2)	992.18 ± 124.88	942.66 ± 74.32	0.731898644
SM(24:3)	101.72 ± 7.72	98.51 ± 6.25	0.74745479
SM(24:4)	15.53 ± 1.03	15.13 ± 1.03	0.782614192
SM(18:2)	2.3 ± 0.26	2.35 ± 0.23	0.880744276
SM(20:1)	76.21 ± 6.25	64.78 ± 7.66	0.260913317
SM(20:2)	5.71 ± 0.4	5.29 ± 0.48	0.508237074
SM(20:3)	2.83 ± 0.24	2.76 ± 0.22	0.847056906
SM(22:1)	869.58 ± 59.41	719.95 ± 64.73	0.100529099
Sum SM	7141.62 ± 450.38	6437.08 ± 478.93	0.294190086
GlcCer(20:0)	122.52 ± 29.39	85.23 ± 29.89	0.381846433
GlcCer(23:0)	59.05 ± 13.2	149.15 ± 31.1	0.01435808
GlcCer(22:0)	138.56 ± 46.88	99.75 ± 33.29	0.500776786
GlcCer(16:0)	65.38 ± 18.38	113.64 ± 24.24	0.127033377
GlcCer(24:0)	147.54 ± 55.95	107.07 ± 26.71	0.51055442
GlcCer(18:0)	84.95 ± 18.08	49 ± 11	0.095546792
GlcCer(24:1)	132.56 ± 48.93	131.95 ± 35.3	0.991894978
Sum GluCer	750.56 ± 122.12	735.8 ± 69.73	0.915793931
LacCer(20:0)	21.53 ± 7.56	22.59 ± 8.04	0.924616231
LacCer(23:0)	94.4 ± 40.48	102.63 ± 36.43	0.880625913
LacCer(18:0)	49.18 ± 18.3	49.81 ± 16.52	0.979846143
SPB(18:1;O2)	60.49 ± 8.32	64.87 ± 9.42	0.731213441
SPB(18:1;O)	34.87 ± 5.97	27.7 ± 4.12	0.325458991
SPBP(18:1;O2)	1.27 ± 0.42	1.42 ± 0.4	0.798705321
Cer(14:0)	14.34 ± 1.62	14.43 ± 2.03	0.973356252
Cer(16:0)	347.62 ± 38.02	348.73 ± 44.38	0.985091962
Cer(18:0)	57.14 ± 6.59	63.16 ± 11.94	0.668464296
Cer(20:0)	14.75 ± 1.8	17.2 ± 3.6	0.556749128
Cer(22:0)	62.28 ± 7.42	68.14 ± 11.45	0.674944302
Cer(23:0)	21.77 ± 2.49	24.38 ± 3.6	0.561168555
Cer(24:0)	117.1 ± 12.27	136.36 ± 20.65	0.436842264
Cer(24:1)	233.01 ± 24.62	264.14 ± 31.36	0.445154927
Cer(24:2)	16.13 ± 1.74	18.84 ± 2.6	0.399043968
Cer(26:0)	1.22 ± 0.17	1.31 ± 0.22	0.736714756
Cer(26:1)	3.59 ± 0.4	3.9 ± 0.48	0.621266779
Sum Cer	888.94 ± 87.16	960.58 ± 125.3	0.646465704
Cer(16:0;O2)	66.9 ± 13.07	46.34 ± 8.61	0.193853799
Cer(18:0;O2)	15.36 ± 3.18	10.89 ± 2.5	0.274588683
Cer(20:0;O2)	12.98 ± 2.99	7.9 ± 1.86	0.154858049
Cer(22:0;O2)	26.01 ± 6	16.28 ± 3.89	0.178765139
Cer(23:0;O2)	6.05 ± 1.43	3.77 ± 0.83	0.172916143
Cer(24:0;O2)	19.29 ± 4.63	12.97 ± 2.74	0.243017852

Cer(26:0;O2)	0.37 ± 0.07	0.28 ± 0.07	0.367725357
Cer(26:1;O2)	1.06 ± 0.25	0.99 ± 0.38	0.884387816
Sum O2Cer	148.02 ± 28.52	99.43 ± 19.2	0.163397458
TG(48:2)	531.15 ± 55.3	575.35 ± 65.07	0.610989048
TG(48:1)	1115.78 ± 96.75	1199.64 ± 107.34	0.567936711
TG(48:0)	547.58 ± 82.46	581.99 ± 70.57	0.752502478
TG(50:3)	605.14 ± 54.77	672.35 ± 73.4	0.473543921
TG(50:2)	2990.27 ± 292.28	3151.69 ± 321.06	0.713910671
TG(50:1)	3551.51 ± 523.05	3739.1 ± 466.58	0.790325681
TG(50:0)	327.61 ± 61.42	358.95 ± 51.27	0.69678307
TG(52:5)	158.78 ± 17.21	203.42 ± 17.78	0.08228194
TG(52:4)	547.98 ± 44.28	644.01 ± 50.73	0.166749458
TG(52:3)	3191.3 ± 312.67	3344.01 ± 356.72	0.751120469
TG(52:2)	8309.02 ± 1025.56	8762.78 ± 991.59	0.752689107
TG(52:1)	1588.84 ± 253.42	1789.34 ± 228.41	0.560359064
TG(52:0)	73.18 ± 14.74	89.71 ± 13.86	0.420217157
TG(54:6)	343.93 ± 37.54	414.03 ± 27.15	0.137510544
TG(54:5)	818.85 ± 80.42	962.02 ± 69.02	0.185427162
TG(54:4)	1508.38 ± 138.63	1660.81 ± 143.17	0.451492849
TG(54:3)	6259.11 ± 659.33	6526.69 ± 730.25	0.788494489
TG(54:2)	2844.56 ± 315.17	3140.98 ± 385.75	0.559422758
TG(54:1)	103.75 ± 19.57	124.61 ± 20.13	0.464495646
TG(56:7)	1095.18 ± 129.09	1267.5 ± 106.35	0.308846983
TG(56:6)	1100.57 ± 112.44	1231.99 ± 103.98	0.397159938
TG(56:5)	1222.35 ± 113.03	1334.77 ± 129.6	0.520736916
TG(56:4)	1494.33 ± 189.31	1615.7 ± 209.71	0.672028194
TG(56:3)	346.08 ± 54.08	374.61 ± 56.63	0.718938495
Sum TG	40675.25 ± 4189.69	43766.03 ± 4285.03	0.610595257
DG(14:0/14:0/0:0)	1.31 ± 0.35	1.18 ± 0.3	0.773131696
DG(14:0/16:1/0:0)	4.55 ± 0.89	4.6 ± 0.89	0.968342636
DG(14:0/16:0/0:0)	22.58 ± 2.97	23.31 ± 3.35	0.872194572
DG(16:1/16:1/0:0)	5.63 ± 0.7	5.64 ± 0.96	0.994871936
DG(14:0/18:1/0:0)	22.22 ± 3.02	22.74 ± 3.01	0.903875839
DG(16:0/16:1/0:0)	58.39 ± 7.32	56.93 ± 6.51	0.882688466
DG(14:0/18:0/0:0)	1.89 ± 0.28	1.9 ± 0.23	0.977878695
DG(16:0/16:0/0:0)	82.91 ± 14.23	77.52 ± 7.24	0.733400233
DG(16:0/18:2/0:0)	17.83 ± 2.17	18.52 ± 2.07	0.82020472
DG(16:1/18:1/0:0)	90.98 ± 9.92	89.28 ± 10.89	0.909709766
DG(16:0/18:1/0:0)	379.02 ± 56.1	374.47 ± 40.32	0.947413203
DG(18:0/16:1/0:0)	20.51 ± 2.12	20.49 ± 1.93	0.996065079
DG(16:0/18:0/0:0)	36.65 ± 6.85	36.49 ± 3.36	0.982811808
DG(16:0/20:4/0:0)	26.16 ± 3.47	25.31 ± 1.68	0.823084543
DG(18:2/18:2/0:0)	0.74 ± 0.11	0.88 ± 0.16	0.501310328
DG(16:0/20:3/0:0)	12.42 ± 1.59	11.79 ± 1.1	0.744396957
DG(18:0/18:3/0:0)	1.1 ± 0.12	1.22 ± 0.15	0.564735419
DG(18:2/18:1/0:0)	30.72 ± 3.42	30.5 ± 3.47	0.964348645
DG(16:0/20:2/0:0)	11.79 ± 2.44	11.34 ± 1.04	0.862559405
DG(18:0/18:2/0:0)	21.6 ± 1.47	19.9 ± 1.92	0.493568118
DG(18:1/18:1/0:0)	306.67 ± 30.47	290.38 ± 33.56	0.723069488
DG(16:0/20:1/0:0)	29.65 ± 5.58	27.62 ± 3.33	0.753453203
DG(18:0/18:1/0:0)	177.49 ± 16.6	168.84 ± 13.47	0.686806912
DG(16:0/20:0/0:0)	2.13 ± 0.65	1.61 ± 0.26	0.45349483
DG(18:0/18:0/0:0)	9.21 ± 0.72	8.79 ± 0.79	0.695522154
DG(16:0/22:6/0:0)	14.64 ± 3.86	14.13 ± 1.37	0.8998661
DG(18:0/20:4/0:0)	266.61 ± 28.05	204.7 ± 19.11	0.075306626
DG(18:0/22:6/0:0)	12.71 ± 1.27	11.79 ± 0.89	0.552834495
DG(14:0/0:0/16:0)	5.96 ± 1.67	7.31 ± 1.38	0.534536649
DG(14:0/0:0/18:1)	5.99 ± 1.36	9.32 ± 2.23	0.221113636
DG(16:0/0:0/16:1)	15.86 ± 3.29	13.61 ± 2.07	0.56216857
DG(16:0/0:0/16:0)	32.63 ± 6.06	23.33 ± 4.09	0.208444705
DG(16:0/0:0/18:2)	8.46 ± 1.69	7.64 ± 1.45	0.714496028
DG(16:1/0:0/18:1)	26.66 ± 3.68	24.63 ± 3.37	0.686657098
DG(16:0/0:0/18:1)	96.14 ± 15.69	105.04 ± 19.66	0.728230691
DG(18:0/0:0/16:1)	6.52 ± 1.58	5.61 ± 2.04	0.727925904

DG(16:0/0:0/18:0)	36.53 ± 5.37	33.87 ± 6.2	0.749255135
DG(16:0/0:0/20:4)	10.51 ± 2.47	13.09 ± 4.12	0.602052092
DG(18:2/0:0/18:1)	13.25 ± 3.71	13.92 ± 2.73	0.885181824
DG(18:0/0:0/18:2)	10.1 ± 2.77	6.99 ± 1.17	0.29833734
DG(18:1/0:0/18:1)	115.02 ± 26.59	84.02 ± 10.1	0.273115175
DG(18:0/0:0/18:1)	57.65 ± 12.12	47.27 ± 9.07	0.494800683
DG(18:0/0:0/18:0)	29.16 ± 4.2	30.63 ± 7.16	0.863118227
DG(16:0/0:0/22:6)	12.63 ± 4.15	9.15 ± 1.86	0.441036385
DG(18:0/0:0/20:4)	164.49 ± 24.43	100.85 ± 18.04	0.043198995
DG(18:0/0:0/22:6)	9.89 ± 1.75	9.72 ± 1.77	0.9456437
Sum DG	2325.57 ± 213.43	2107.83 ± 175.19	0.434301039

FARF condition

Feature	NW, mean ± SEM	Ob, mean ± SEM	p.value
CAR(12:0)	1.16 ± 0.24	0.82 ± 0.15	0.232069187
CAR(14:0)	5.24 ± 1.29	3.11 ± 0.55	0.130953133
CAR(16:0)	18.07 ± 2.87	13.51 ± 2.31	0.22305452
CAR(16:1)	4.93 ± 0.86	3.02 ± 0.43	0.052930914
CAR(18:0)	12.5 ± 3.12	9.4 ± 2.19	0.417768402
CAR(18:1)	19.3 ± 3.47	13.22 ± 2.38	0.154845417
Sum CAR	61.2 ± 11.09	43.09 ± 7.36	0.179037727
SM(14:0)	664.96 ± 39.48	603.88 ± 39.02	0.280598442
SM(16:1)	278.46 ± 23.83	289.12 ± 25.29	0.761869952
SM(18:1)	88.28 ± 8.81	82.42 ± 9.4	0.653861581
SM(18:0)	775.07 ± 67.94	620.89 ± 87.18	0.177475615
SM(22:0)	1133.16 ± 84.81	913.89 ± 128.66	0.1712229
SM(20:0)	287.51 ± 23.99	230.74 ± 33.99	0.188092997
SM(23:0)	341.6 ± 26.33	312.27 ± 38.52	0.539825266
SM(24:0)	1573.69 ± 134.65	1388.21 ± 158.23	0.382329043
SM(24:2)	975.96 ± 102.47	984.51 ± 109.38	0.955054958
SM(24:3)	98.32 ± 6.71	93.27 ± 8.65	0.651348346
SM(24:4)	14.94 ± 1.14	15.06 ± 1.03	0.935119171
SM(18:2)	2.46 ± 0.21	2.02 ± 0.21	0.153618951
SM(20:1)	76.56 ± 6.55	60 ± 8.15	0.127046556
SM(20:2)	5.27 ± 0.29	4.59 ± 0.51	0.267845282
SM(20:3)	3.04 ± 0.32	2.73 ± 0.28	0.473149741
SM(22:1)	890.26 ± 59.33	719.94 ± 81.6	0.105828547
Sum SM	7209.53 ± 467.24	6323.57 ± 643.71	0.279810054
GlcCer(20:0)	149.73 ± 34.55	89.28 ± 23.35	0.153284548
GlcCer(23:0)	59.38 ± 14.17	131.23 ± 44.1	0.142108807
GlcCer(22:0)	143.17 ± 52.03	127.77 ± 35.43	0.806280398
GlcCer(16:0)	79.47 ± 16.62	92.97 ± 22.78	0.639534132
GlcCer(24:0)	114.35 ± 48.28	116.79 ± 25.87	0.964104555
GlcCer(18:0)	126.01 ± 21.05	49.62 ± 9.11	0.001952486
GlcCer(24:1)	76.83 ± 21.87	115.86 ± 33.68	0.346073968
Sum GluCer	748.94 ± 104.84	723.51 ± 75.6	0.843927624
LacCer(20:0)	22.04 ± 7.88	17.61 ± 7.25	0.681298602
LacCer(23:0)	100.75 ± 45.86	79.53 ± 30.81	0.700517402
LacCer(18:0)	51.86 ± 20.22	38.36 ± 15.1	0.593634915
SPB(18:1;O2)	73.21 ± 8.39	60.45 ± 6.83	0.24498236
SPB(18:1;O)	24.63 ± 3.77	21.16 ± 3.14	0.482141581
SPBP(18:1;O2)	1.96 ± 0.59	1.58 ± 0.35	0.576444008
Cer(14:0)	15.02 ± 1.34	12.7 ± 1.52	0.263326165
Cer(16:0)	344.33 ± 41.46	306.13 ± 42.34	0.524988065
Cer(18:0)	60.16 ± 7.32	54.9 ± 12.11	0.717176157
Cer(20:0)	16 ± 2.13	14.15 ± 3.36	0.650183368
Cer(22:0)	63.59 ± 9.12	60.15 ± 12.15	0.824214844
Cer(23:0)	22.13 ± 3.1	20.04 ± 3.54	0.661404311
Cer(24:0)	120.94 ± 13.62	110.97 ± 18.23	0.667918865
Cer(24:1)	235.43 ± 30.58	226.03 ± 30.7	0.830009452
Cer(24:2)	15.86 ± 2.04	15.41 ± 2.23	0.884331881
Cer(26:0)	1.45 ± 0.18	1.11 ± 0.18	0.190532772
Cer(26:1)	3.52 ± 0.38	3.56 ± 0.46	0.950028556
Sum Cer	898.43 ± 104.67	825.15 ± 121.2	0.652545273

Cer(16:0;O2)	74.7 ± 14.78	51.69 ± 10.33	0.207499247
Cer(18:0;O2)	19.68 ± 4.04	12.18 ± 3.23	0.154667421
Cer(20:0;O2)	15.66 ± 3.38	9.48 ± 2.5	0.148918745
Cer(22:0;O2)	33.03 ± 7.18	18.38 ± 5.03	0.102136784
Cer(23:0;O2)	7.78 ± 1.73	4.07 ± 1.15	0.081345878
Cer(24:0;O2)	26.31 ± 5.51	15.16 ± 4.61	0.129816994
Cer(26:0;O2)	0.43 ± 0.07	0.35 ± 0.1	0.48224472
Cer(26:1;O2)	1.33 ± 0.26	1.03 ± 0.32	0.467908401
Sum O2Cer	178.93 ± 33.17	112.35 ± 24.88	0.116171538
TG(48:2)	397.68 ± 31.13	492.8 ± 46.97	0.106751202
TG(48:1)	754.44 ± 41.97	897.35 ± 98.54	0.202815495
TG(48:0)	339.8 ± 43.83	390.07 ± 55.29	0.485609188
TG(50:3)	480.19 ± 42.03	560.64 ± 54.24	0.254882781
TG(50:2)	1898.84 ± 170.39	2273.85 ± 250.57	0.231772063
TG(50:1)	1933.64 ± 248.62	2289.53 ± 341.11	0.411231096
TG(50:0)	256.84 ± 54.79	284.88 ± 49.25	0.70558552
TG(52:5)	163.42 ± 13.85	251.92 ± 42.96	0.066374851
TG(52:4)	462.61 ± 41.62	723.26 ± 192.12	0.2085986
TG(52:3)	2123.37 ± 216.95	2505.8 ± 345.42	0.363487655
TG(52:2)	4730.55 ± 575.34	5443.75 ± 775.04	0.470612255
TG(52:1)	1094.11 ± 169.27	1431.77 ± 248.57	0.276901843
TG(52:0)	84.74 ± 23.07	104.18 ± 22.83	0.554055637
TG(54:6)	317.57 ± 34.29	582.84 ± 190.47	0.19442692
TG(54:5)	645.63 ± 77.05	1090.46 ± 291.9	0.163129889
TG(54:4)	1178.36 ± 110.67	1653.71 ± 385.01	0.258245594
TG(54:3)	3912.9 ± 427	4508.83 ± 687.08	0.47411533
TG(54:2)	2013.53 ± 197.69	2386.68 ± 370.72	0.390818048
TG(54:1)	86.45 ± 13.73	134.83 ± 27.67	0.135944021
TG(56:7)	892.31 ± 105.7	1127.95 ± 129.78	0.173034173
TG(56:6)	876.13 ± 88.26	1052.82 ± 117.05	0.24249472
TG(56:5)	932.22 ± 82.71	1071.69 ± 125.65	0.36814766
TG(56:4)	1126.98 ± 121.46	1268.2 ± 196.28	0.551681063
TG(56:3)	284.95 ± 36.73	351.03 ± 69.03	0.413971432
Sum TG	26987.28 ± 2664.96	32878.81 ± 4634.73	0.287820333
DG(14:0/14:0/0:0)	1.54 ± 0.6	1.05 ± 0.25	0.445827438
DG(14:0/16:1/0:0)	5.27 ± 1.21	4.4 ± 0.77	0.546331703
DG(14:0/16:0/0:0)	25.21 ± 3.82	19.83 ± 2.24	0.227311037
DG(16:1/16:1/0:0)	5.74 ± 0.92	5.09 ± 0.59	0.546479413
DG(14:0/18:1/0:0)	22.11 ± 3.39	19.38 ± 2.15	0.496171272
DG(16:0/16:1/0:0)	53.86 ± 5.07	46.72 ± 4.06	0.277690174
DG(14:0/18:0/0:0)	2.13 ± 0.32	2.12 ± 0.24	0.971379771
DG(16:0/16:0/0:0)	65.08 ± 5.52	55.98 ± 4.3	0.200370818
DG(16:0/18:2/0:0)	18.49 ± 1.94	17.85 ± 2.06	0.822787582
DG(16:1/18:1/0:0)	80.06 ± 7.34	74.88 ± 6.54	0.601120062
DG(16:0/18:1/0:0)	285.7 ± 27.78	281.01 ± 30	0.909710516
DG(18:0/16:1/0:0)	21.26 ± 2.37	19.23 ± 1.27	0.448846222
DG(16:0/18:0/0:0)	31.68 ± 4.91	30.45 ± 3.1	0.832007905
DG(16:0/20:4/0:0)	25.34 ± 2.57	22.09 ± 1.28	0.257468535
DG(18:2/18:2/0:0)	0.88 ± 0.16	2.7 ± 1.98	0.383558515
DG(16:0/20:3/0:0)	10.63 ± 0.87	10.27 ± 0.77	0.758279602
DG(18:0/18:3/0:0)	1.08 ± 0.11	1.26 ± 0.11	0.257536317
DG(18:2/18:1/0:0)	32.5 ± 3.2	30.95 ± 3.45	0.745188297
DG(16:0/20:2/0:0)	8.36 ± 0.97	8.17 ± 0.66	0.870951243
DG(18:0/18:2/0:0)	22.1 ± 1.28	19.85 ± 0.97	0.16799099
DG(18:1/18:1/0:0)	236.72 ± 21.37	212.42 ± 14.31	0.346984689
DG(16:0/20:1/0:0)	22.94 ± 4.52	21.09 ± 2.33	0.712897171
DG(18:0/18:1/0:0)	163.43 ± 14.04	150 ± 7.63	0.39956922
DG(16:0/20:0/0:0)	1.87 ± 0.36	1.68 ± 0.19	0.641310111
DG(18:0/18:0/0:0)	8.3 ± 0.79	7.6 ± 0.61	0.484595546
DG(16:0/22:6/0:0)	14.97 ± 2.87	13.45 ± 1.1	0.617183369
DG(18:0/20:4/0:0)	239.66 ± 21.81	191.84 ± 19.36	0.110912425
DG(18:0/22:6/0:0)	11.03 ± 0.73	10.96 ± 0.87	0.948550872
DG(14:0/0:0/16:0)	7.81 ± 1.32	7.3 ± 1.39	0.793239788
DG(14:0/0:0/18:1)	5.77 ± 1.56	5.13 ± 1.14	0.738284078

DG(16:0/0:0/16:1)	18.81 ± 3.04	16.52 ± 2.56	0.56567755
DG(16:0/0:0/16:0)	20.02 ± 3.91	20.81 ± 3.08	0.874090343
DG(16:0/0:0/18:2)	5.56 ± 0.95	10.13 ± 2.53	0.109635715
DG(16:1/0:0/18:1)	17.56 ± 3.43	28.78 ± 7.65	0.200709381
DG(16:0/0:0/18:1)	84.39 ± 13.61	70.36 ± 12.45	0.452354441
DG(18:0/0:0/16:1)	5.13 ± 1.27	5.4 ± 1.13	0.873549758
DG(16:0/0:0/18:0)	30.55 ± 5.61	31.61 ± 7.73	0.913559203
DG(16:0/0:0/20:4)	9.06 ± 1.69	7.37 ± 1.53	0.465716702
DG(18:2/0:0/18:1)	12.22 ± 2.66	13.74 ± 4.47	0.775700899
DG(18:0/0:0/18:2)	8.56 ± 1.78	11.69 ± 3.7	0.461910405
DG(18:1/0:0/18:1)	78.2 ± 11.96	61.91 ± 8.97	0.280882358
DG(18:0/0:0/18:1)	51.6 ± 11.62	47.07 ± 8.41	0.752445203
DG(18:0/0:0/18:0)	28.55 ± 5.83	38.14 ± 12.28	0.495302434
DG(16:0/0:0/22:6)	11.22 ± 5.01	9.44 ± 1.79	0.734126025
DG(18:0/0:0/20:4)	151.81 ± 27.54	99.38 ± 24.79	0.166633203
DG(18:0/0:0/22:6)	10.56 ± 2.12	6.02 ± 1.05	0.059820253
Sum DG	1975.32 ± 159.57	1773.12 ± 137.58	0.343302455
